# Freestanding CuBTC‐Doped Fluorine‐Containing Flexible Membrane with Ultrafast Oil‐Water Separation via Enhanced Electrostatic Repulsion Force

**DOI:** 10.1002/advs.202509657

**Published:** 2025-09-14

**Authors:** Xiaoshuang Li, Peize Yang, Minghui Zhu, Zhen Zhang, Boyu Lei, Bing Geng, Guanghui Cui, Mei Yan

**Affiliations:** ^1^ School of Chemistry and Chemical Engineering University of Jinan Jinan 250022 China; ^2^ Shandong Provincial Key Laboratory of Fluorine Chemistry and Chemical Materials University of Jinan Jinan 250022 China; ^3^ Drilling Fluid Research Center of Mud Service Branch Bohai Drilling Engineer Company China National Petroleum Corporation (CNPC) Tianjin 300280 China

**Keywords:** electrostatic repulsion force, flexible membrane, high internal phase emulsion, MOFs, oil/water separation

## Abstract

To address the challenge of uneven distribution of metal–organic frameworks (MOFs) within the membrane in oil‐water separation, this study demonstrates a novel copper (II) benzene‐1,3,5‐tricarboxylate (CuBTC) embedded fluorinated composite membrane fabricated through high internal phase emulsion (HIPE) templating. During polymerization, MOFs spontaneously align at porous matrix interfaces via interfacial confinement, effectively suppressing particle aggregation. The resultant MOF‐polymer composite membranes exhibit some critical advantages: ultrafast oil‐water separation kinetics (60,854.4 L·m^−^^2^·h^−1^·bar^−1^ for n‐hexane); chemical stability in extreme pH environments (pH 1–12). Molecular dynamics simulations revealed a unique separation mechanism dominated by synergistic electrostatic repulsion‐based CuBTC and the fluoro‐polymer matrix, challenging the conventional requirement of superhydrophobicity for efficient separation. The optimized membrane achieved 99.4% separation efficiency with <2% performance decay over 20 cycles. Furthermore, a universal flux‐thickness correlation (*J*  =  *Ae* ^−*T*/*B*^ + *C*) enabling quantitative comparison of separation membranes independent of thickness variations is established.

## Graphical abstract

A new type of CuBTC‐Doped Fluorene‐Containing Flexible Membrane (CuBTCFFM) was rapidly prepared by high internal phase emulsion template method and ultraviolet polymerization process, which has excellent oil‐water separation efficiency and high permeate flux. The separation mechanism of membrane is dominated by strong electrostatic repulsion based on CuBTC and fluoropolymer matrix.

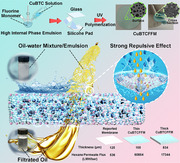

## 1 Introduction

Membrane separation has emerged as a viable solution for large‐scale treatment of surfactant‐stabilized oil‐in‐water emulsions prevalent in petrochemical refining and metalworking.^[^ ^1^, ^2^, ^3^ ^]^ Early‐stage membrane separation materials were predominantly fabricated through size‐exclusion mechanisms, with supplementary oil‐droplet removal achieved via synergistic interactions of retention, inertial impaction, and pore‐size discrimination within the filtration matrix.^[^ ^4^ ^]^ Building upon these findings, researchers further have manufactured superwetting substrates with contrasting liquid affinities, achieving excellent separation efficiency through selective permeability mechanisms.^[^ ^5^, ^6^, ^7^ ^]^ Polymer membranes can be rationally designed to achieve simultaneous optimization of permeate flux and fouling resistance.

Since their structural engineering breakthrough in 2016, metal‐organic frameworks (MOFs)—porous coordination polymers formed through metal‐cluster and organic‐linker assembly—have emerged as promising candidates for advanced oil‐water separation technologies.^[^ ^8^, ^9^, ^10^ ^]^ The structural tunability of MOFs—encompassing precisely engineered pore geometries, customizable surface functionalities, and ultrahigh porosity, endow MOFs with tunable wetting properties.^[^ ^11^, ^12^, ^13^ ^]^ CuBTC, known as HKUST‐1 or MOF‐199, was first reported by Williams' research group in 1999. As one of the most representative MOFs, it possesses abundant unsaturated metal sites.^[^ ^14^, ^15^ ^]^ To enable the application of CuBTC in oil‐water separation, its wettability must be modified through strategies such as deposition onto a hydrophobic substrate or post‐treatment with low surface energy materials in order to construct mixed matrix membranes (MMMs).^[^ ^16^, ^17^ ^]^ Nevertheless, MOF‐MMMs continue to exhibit critical limitations in some key operational aspects: sustained durability, environmental resilience, and chemical resistance.^[^ ^18^ ^]^ Furthermore, the preparation process of MOF‐MMMs is relatively intricate, its mechanical properties are constrained, and the pore size is susceptible to blockage, thereby resulting in a comparatively low permeate flux.^[^ ^19^ ^]^ The strong polarity of C─F bonds in fluoropolymers makes them ideal matrix materials for MOF‐incorporated mixed matrix membranes, providing both superior hydrophobicity and acid/alkali resistance.^[^ ^20^, ^21^ ^]^

The fabrication of MOF‐MMMs demands precise control over both the dispersion homogeneity and spatial arrangement of MOF particles within the polymer matrix. Conventional dispersion techniques, including high‐shear mixing, ultrasonication, and direct coating, often result in nanoparticle aggregation and heterogeneous dispersion due to inherent interfacial incompatibility between the metal–organic framework (MOF) fillers and the polymer matrix.^[^ ^22^, ^23^ ^]^ This phenomenon primarily stems from mismatched surface energetics at the organic‐inorganic phase boundaries.^[^ ^24^ ^]^ In comparison, the high internal phase emulsion (HIPE) templating approach exploits interfacial confinement effects at oil‐water biphasic interfaces.^[^ ^25^ ^]^ These effects drive the thermodynamically favored alignment of MOF particles along the pore walls of fluorinated polymer membranes during phase inversion, thereby achieving guaranteed macroscopic dispersibility.^[^ ^26^ ^]^ The combination of MOF particles and fluorinated polymers enables a synergistic enhancement in both the electronegativity of MOFs and the polarity of the fluorinated polymer.^[^ ^27^ ^]^ The interfacial polarization effect induces a pronounced electrostatic repulsive force that effectively repels polar water molecules upon contact with fluorinated MOF membranes, thereby enabling the efficient separation of oil‐water mixtures.^[^ ^28^, ^29^ ^]^ This mechanism operates through a fundamentally distinct pathway compared to conventional size‐exclusion and surface‐wettability paradigms, yet its underlying principles and scalability remain inadequately explored in the existing literature.

**Figure 1:**
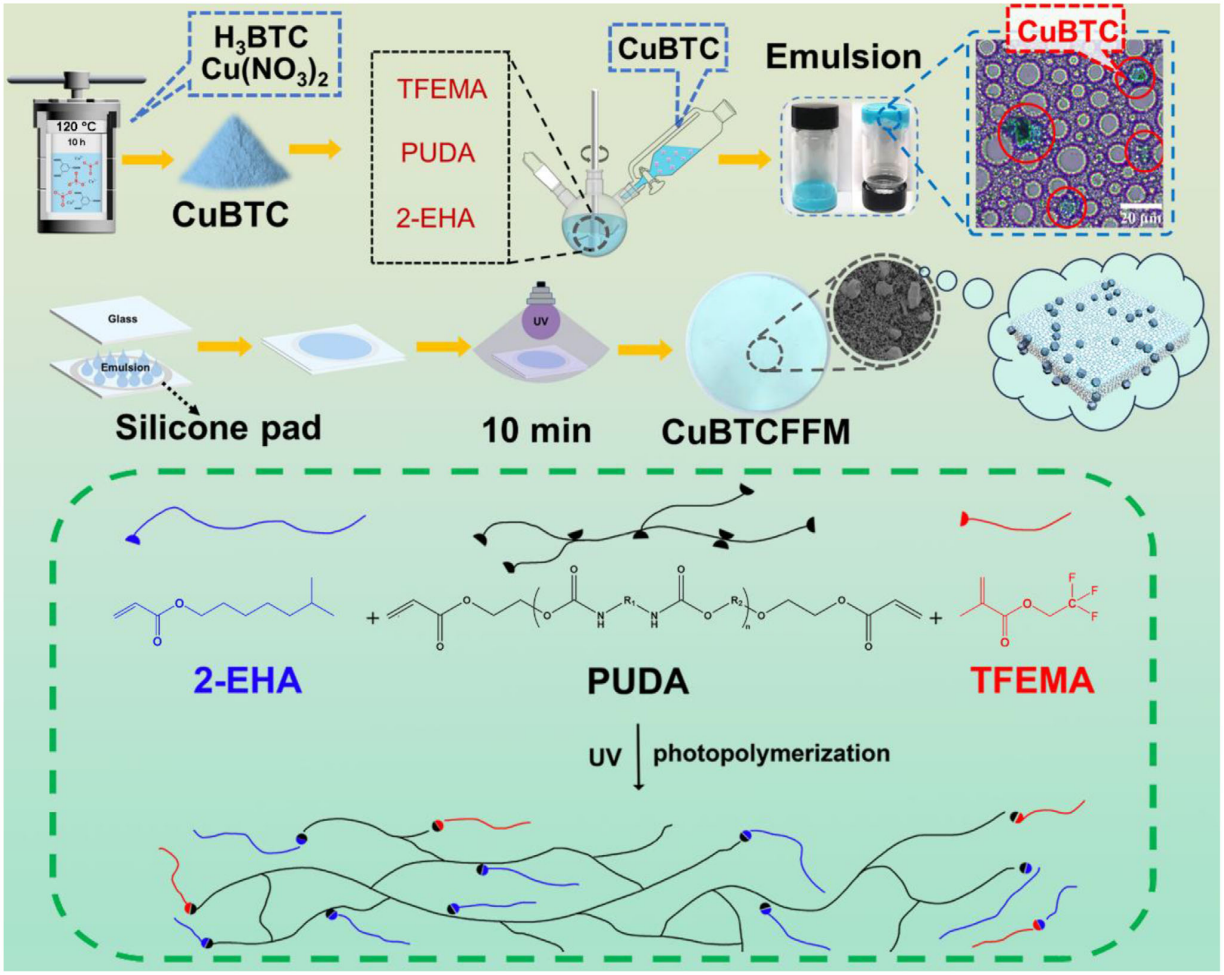
Fabrication of the CuBTC‐doped fluorine‐containing flexible membrane (CuBTCFFM).

In this study, a high internal phase emulsion (HIPE) templating strategy was applied for fabricating flexible MOF composite membranes with architecturally tunable porosity. The methodology integrates a ternary monomer system comprising: i) trifluoroethyl methacrylate (TFEMA) to establish hydrophobicity, ii) 2‐ethylhexyl acrylate (2‐EHA) as a chain‐flexibilizing comonomer, iii) polyurethane diacrylate (PUDA) serving as a crosslinker for network stabilization. The HIPE‐templated interfacial confinement dynamically mediated the in situ coordination synthesis of copper (II) benzene‐1,3,5‐tricarboxylate (CuBTC) particles, enabling precise incorporation of CuBTC particles as functional fillers with homogeneous dispersion (**Figure** **1**). The prepared fluoropolymer matrix confers UV‐weathering resistance to the membrane while inducing moderate hydrophobicity (water contact angle: 120°‐135°), falling short of the superhydrophobic threshold (WCA >150°). The HIPE‐derived membranes exhibited a hierarchically porous architecture with a well‐defined pore size distribution centered at 1 µm, directly ensuring an ultrahigh permeate flux (60854.4 L·m^−2^·h^−1^·bar^−1^ for n‐hexane), representing some of the highest values recorded among existing mixed matrix membranes. Crucially, we establish a previously unrecognized exponential correlation between permeate flux and membrane thickness. Despite the absence of conventional size‐exclusion mechanisms and surface‐wettability‐based separation principles in this research, the developed MOF‐fluoropolymer composite membranes exhibited exceptional separation efficiency (>99.4%) for both oil‐water mixtures and emulsions. This counterintuitive performance unambiguously reveals a novel separation mechanism dominated by the synergistic enhancement of polarity‐derived repulsive forces of the MOF‐fluorinated polymer interface, as evidenced by molecular dynamics simulations.

## 2 Results and Discussion

### 2.1 Structures and Properties of CuBTCFFM

**Figure 2:**
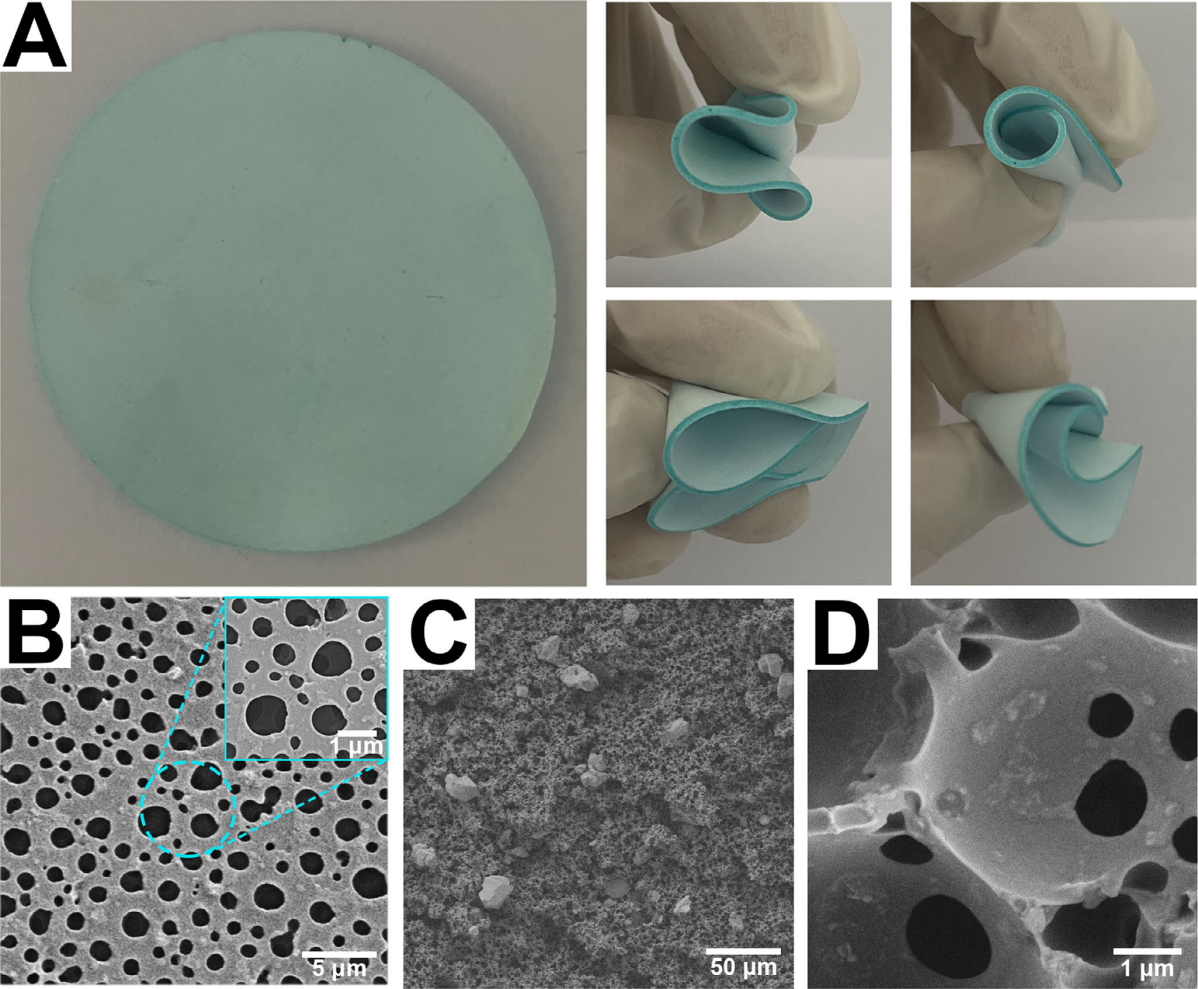
A) Appearance and bending flexibility, (B) the surface, and (C and D) the cross‐sectional SEM images with different magnifications of the CuBTCFFM.

CuBTC was synthesized using the hydrothermal method,^[^ ^30^ ^]^ as shown in Figure 1. The XRD analysis (Figure S1, Supporting Information) shows that the synthesized CuBTC has the cub‐octahedral structure. The fluorinated acrylate emulsion can be photopolymerized in a short time under the irradiation of a UV lamp, and the MOFs‐fluoropolymer mixed‐matrix membrane can be obtained after drying, using the high internal phase emulsion template method. A membrane with a diameter of 5 cm was obtained by clipping (**Figure** **2**A). The membrane has certain flexibility and can be bended easily. The morphology of the CuBTCFFM mixed‐matrix membrane was analyzed by SEM and EDS elemental diagram. Figure 2B shows that the CuBTCFFM has a uniform porous structure, with through holes of ≈ 1 µm in diameter. Figure 2C,D are the cross section of CuBTCFFM, and the thickness of the membrane is about 975 µm. Figure 2D shows that the membrane retains a through‐hole internal structure, which serves as a foundation for oil‐water filtration and separation. Figure 2D shows that CuBTC nanoparticles with size of 50 to 200 nm are distributed on the wall surface of the holes. However, it should be acknowledged that during the preparation process, some CuBTC formed large aggregates, which were also embedded in the matrix—though not within the holes (Figure 2C). In short, these results verify the successful embedding of MOFs in the membrane.

**Figure 3:**
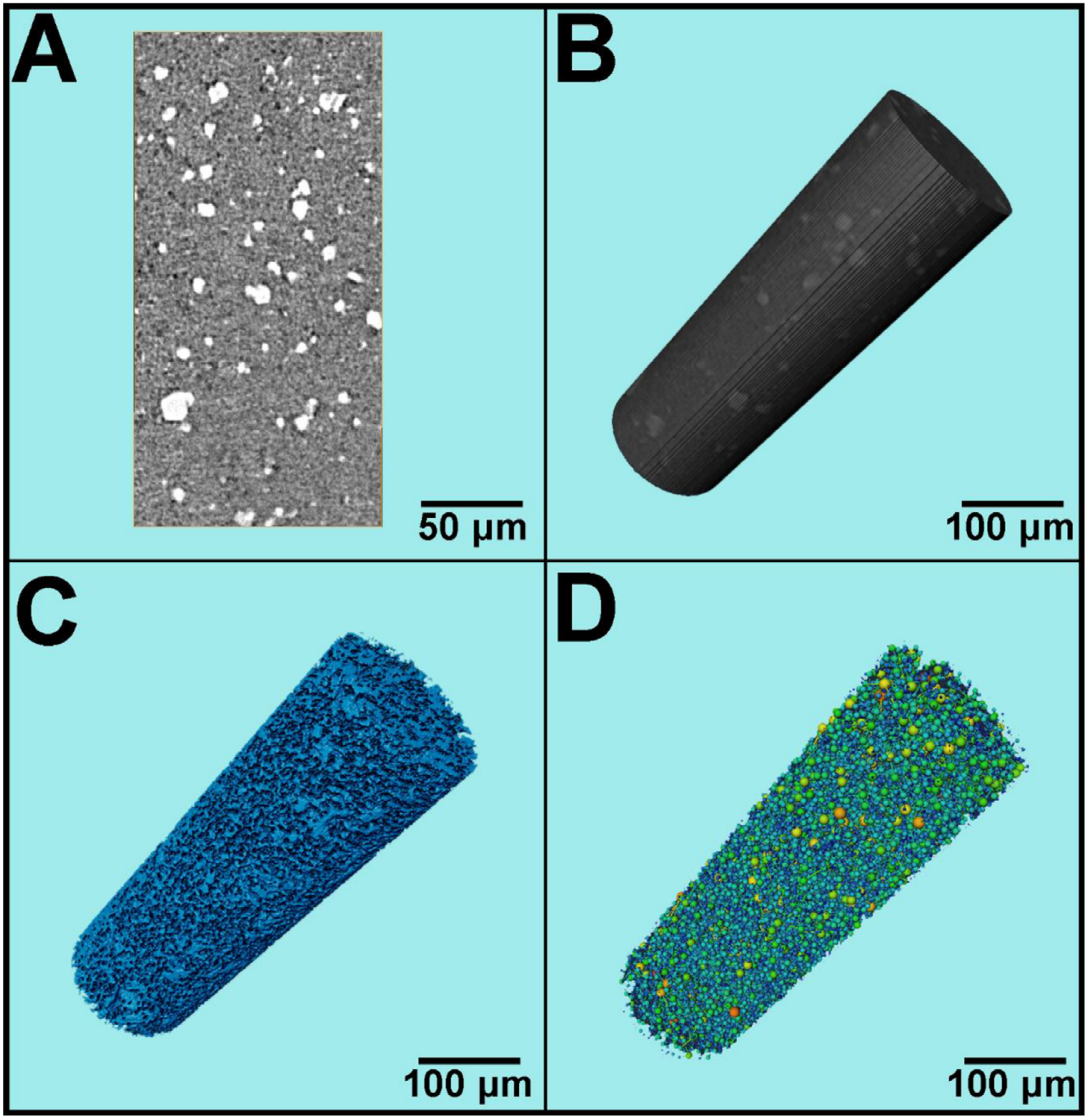
A) X‐CT 2D cross‐sectional image, B) 3D simulation reconstruction image, C) Pore extraction diagram, and D) Generated pore network model of CuBTCFFM. The white regions in (A) represent CuBTC.

X‐CT is a non‐destructive method used for 3D characterization imaging. The sample is rotated slowly on a sample stage, with angle adjusted to capture 2D X‐ray images from various perspectives. A large number of slices are acquired, which are processed and reconstructed into 3D simulated structures of the sample using Avizo software. The cross‐sectional tomography and simulated 3D images (**Figure** **3**A) demonstrate that the grey and black regions correspond to the framework and macropores of the membrane, respectively, while the white color regions represent CuBTC. Figure 3B is the 3D simulation reconstruction image of CuBTCFFM. These images confirm the medium‐homogeneous distribution of CuBTC in the polymer membrane. The interactive thresholding command was used to extract the pores, and a 3D pore structure map was generated (Figure 3C). The connected pore structure of the membrane was calculated using the Axis Connectivity command. Subsequently, a pore network model was constructed (Figure 3D). The model uses colored balls to differentiate between pore size ranges, and through pores are connected by ball and stick. Based on this model, the porosity of the CuBTCFFM membrane was calculated to be ≈15.42%. Both the SEM (Figure 2) and 3D images (Figure 3) reveal that the CuBTCFFM has macroporous channels that could facilitate the transportation of water and oil.

**Figure 4:**
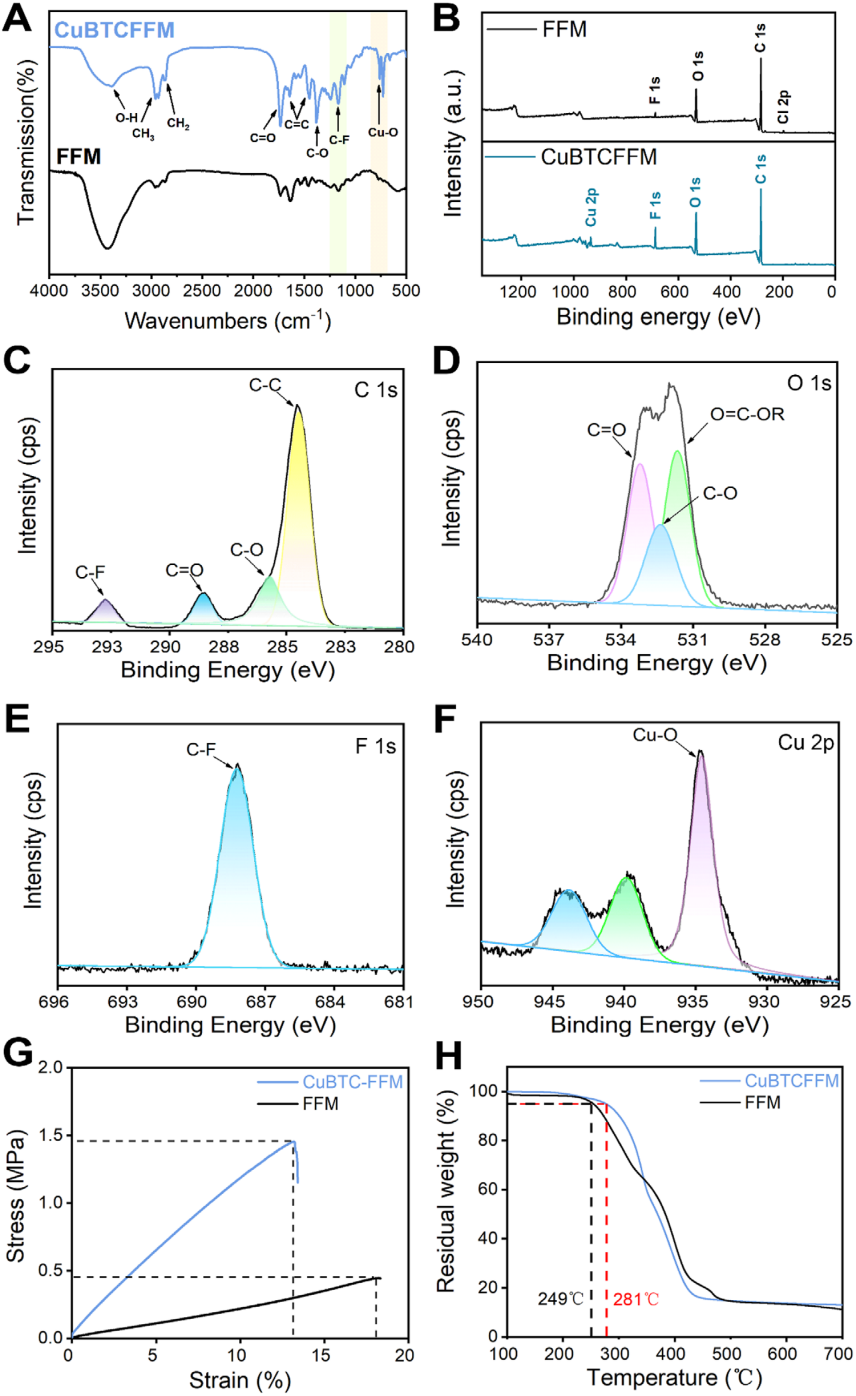
A) The ATR‐FTIR spectra and B) XPS full spectrum of the FFM and CuBTCFFM. High‐resolution XPS spectrum of CuBTCFFM: C) C 1s, D) O 1s, E) F 1s, and F) Cu 2p. G) stress‐strain curves and H) thermal stability curves of FFM and CuBTCFFM.

To evaluate whether the MOFs were successfully grafted onto the surface of the membrane, the prepared CuBTCFFM with different MOFs content were characterized by ATR‐FTIR (**Figure** **4**A). By comparing the infrared spectra of FFM and CuBTCFFM, the stretching Cu─O vibration was observed at the wavenumber 730 cm^−1^ in the CuBTCFFM, and the intensity of the peak gradually increased with the increase of CuBTC content. These results proves that CuBTC was successfully incorporated into the membrane using the high internal phase emulsion template method. The strong absorption peaks at 1375, 1450, and 1641 cm^−1^ indicate the presence of the stretching vibration of the C─O group, the aromatic C═C vibration and the stretching vibration of the C═O group, while the broad band at 3400 cm^−1^ indicates the presence of the vibration of the O─H group. In addition, the absorption peak at 1200 cm^−1^ corresponds to the stretching vibration of the C─F group, which proves that the high internal phase emulsion was successfully prepared as a fluoropolymer‐based polymer by photoinitiated polymerization.^[^ ^31^ ^]^ The elemental distribution on the surface of the membrane was further analyzed using X‐ray photoelectron spectroscopy. Figure 4B shows the complete XPS spectra of the blank FFM and the CuBTCFFM.^[^ ^30^ ^]^ The comparison shows that when the aqueous phase is replaced from the aqueous calcium chloride solution to the aqueous solution of CuBTC containing copper ions, the disappearance of the organic Cl binding energy peak of 198.9 eV and the formation of the Cu─O peak at 934.6 eV can be clearly observed. The elemental composition of CuBTCFFM was further analyzed based on the high‐resolution spectrum of the elements (Figure 4C–F), and energy spectrum peaks of C 1s, O 1s, F 1s, and Cu 2p were detected in the synthesized CuBTCFFM sample. In addition, the electron binding energy spectrum of C 1s can be divided into three peaks at 284.8, 285.4, and 289.2 eV (Figure 4C), which correspond to the C─C, C═O, and C─F binding energy peak, respectively. The binding energy peak at 531.8 eV in the electron binding energy spectrum of O 1s is attributed to the ester oxygen of O═C─OR, proving the formation of an aromatic polyester (Figure 4D). The peak at 688.1 eV proves the presence of a C─F bond (Figure 4E). The Cu─O bond energy peak at 934.6 eV and two satellite peaks at 934.7 and 945.2 eV in Figure 4F prove the existence of CuBTC, indicating that CuBTC is successfully distributed on the surface of the membrane.^[^ ^32^ ^]^ In short, CuBTC‐doped fluoropolymer mixed‐matrix membranes are successfully fabricated.

Unlike other rigid membranes that use stainless steel mesh or copper mesh as the substrate,^[^ ^16^, ^33^ ^]^ the CuBTCFFM exhibits hyperplastic properties with large deformations (Figure 4G).The membrane has excellent tensile properties, which can be easily bent and can restored to original shape quickly. Comparing the stress‐strain curves of the FFM and CuBTCFFM shows that CuBTCFFM, has higher tensile strength and fracture stress, while CuBTCFFM, breaks at a lower load. This is attributed to the doping and embedding of MOFs within the substrate, which improves the mechanical strength of the interface between the MOFs and the polymer matrix. The stronger tensile strength of CuBTCFFM helps the membrane to withstand higher vacuum degree during the filtration process. The thermal weight loss (TGA) method was chosen to evaluate the thermal stability of the fabricated CuBTCFFM. As shown in Figure 4H, the thermal decomposition temperature of the CuBTCFFM is 281 °C at 5 wt.% weight loss, which is 32 °C higher than that of FFM. This is due to the porous skeleton structure of the MOFs with higher thermal stability. However, MOFs in the CuBTCFFM decomposes, when the temperature exceeds 350 °C. The filtrate temperature in industrial production normally does not exceed 250 °C. Thus, the CuBTCFFM with good thermal stability can be used as a high‐temperature resistant separation membrane.

**Figure 5:**
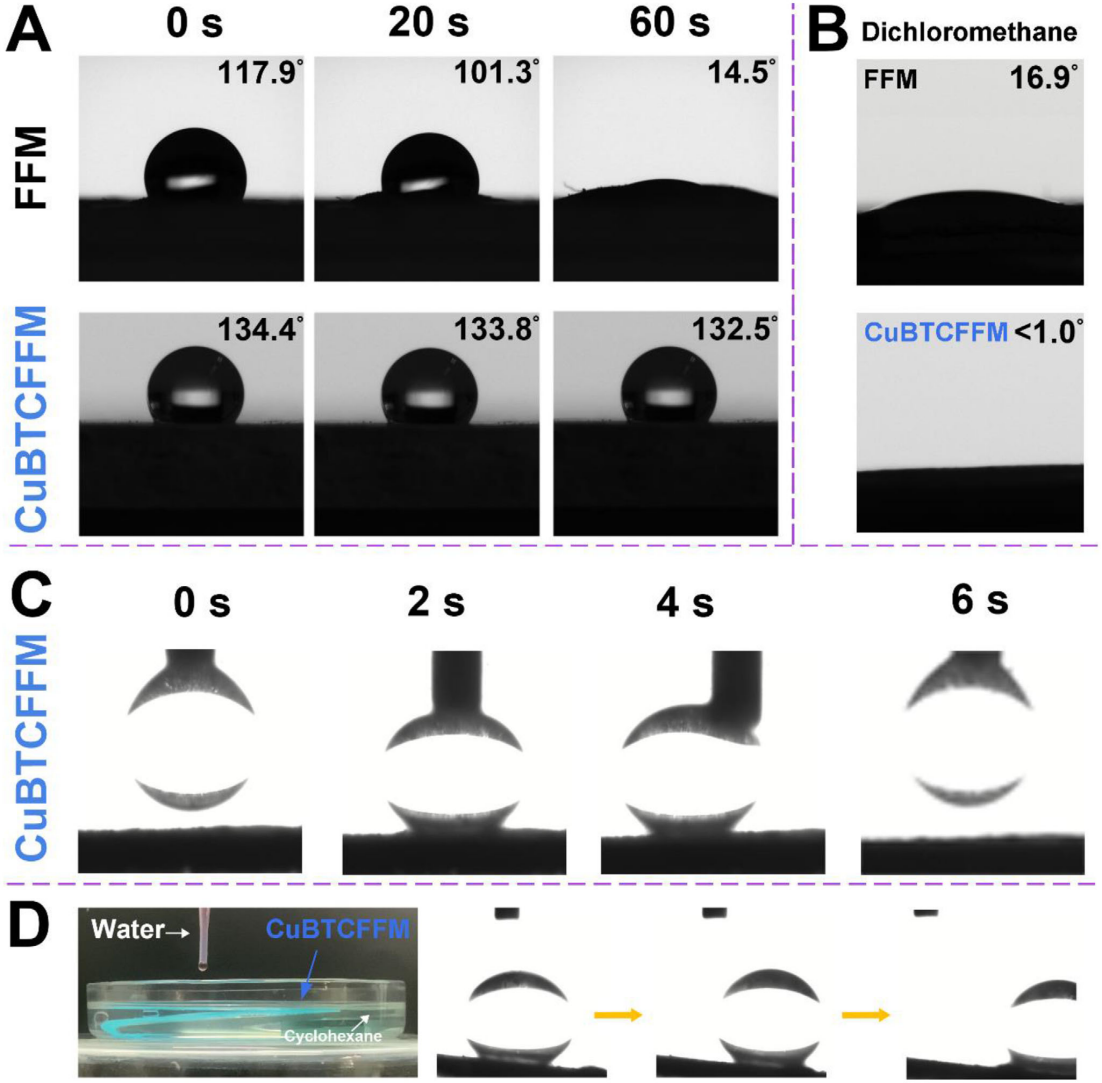
A) Water contact angles on FFM and CuBTCFFM at different time, B) Oil contact angles on FFM and CuBTCFFM, C) Under‐oil water contact angle, and D) superhydrophobic rolling behavior of under‐oil water droplets of CuBTCFFM.

The water contact angles (WCA), oil contact angles (OCA) and under‐oil water contact angle (UOWCA) on FFM and CuBTCFFM were tested (**Figure** **5**). The water contact angle on FFM is 117.9°, which increases to ≈ 134° on CuBTCFFM, indicating that the addition of MOFs can improve hydrophobicity (Figure 5A). Over time, the water contact angle on FFM gradually decreased to 14.5°, and the slow transition from hydrophobicity to hydrophilicity hinders the effectiveness in oil‐water separation applications. While the contact angle on CuBTCFFM remained almost unchanged, and the sustained hydrophobicity of CuBTCFFM provides a fundamental assurance for the suitability in oil‐water separation. The contact angles of FFM and CuBTCFFM to dimethylene chloride were also tested (Figure 5B–D). The measurements of the oil contact angle and water contact angle indicate that CuBTCFFM exhibits oleophilic and hydrophobic properties (Movie S1–S4, Supporting Information).

### 2.2 Permeability and Separation Performance of CuBTCFFM

**Figure 6:**
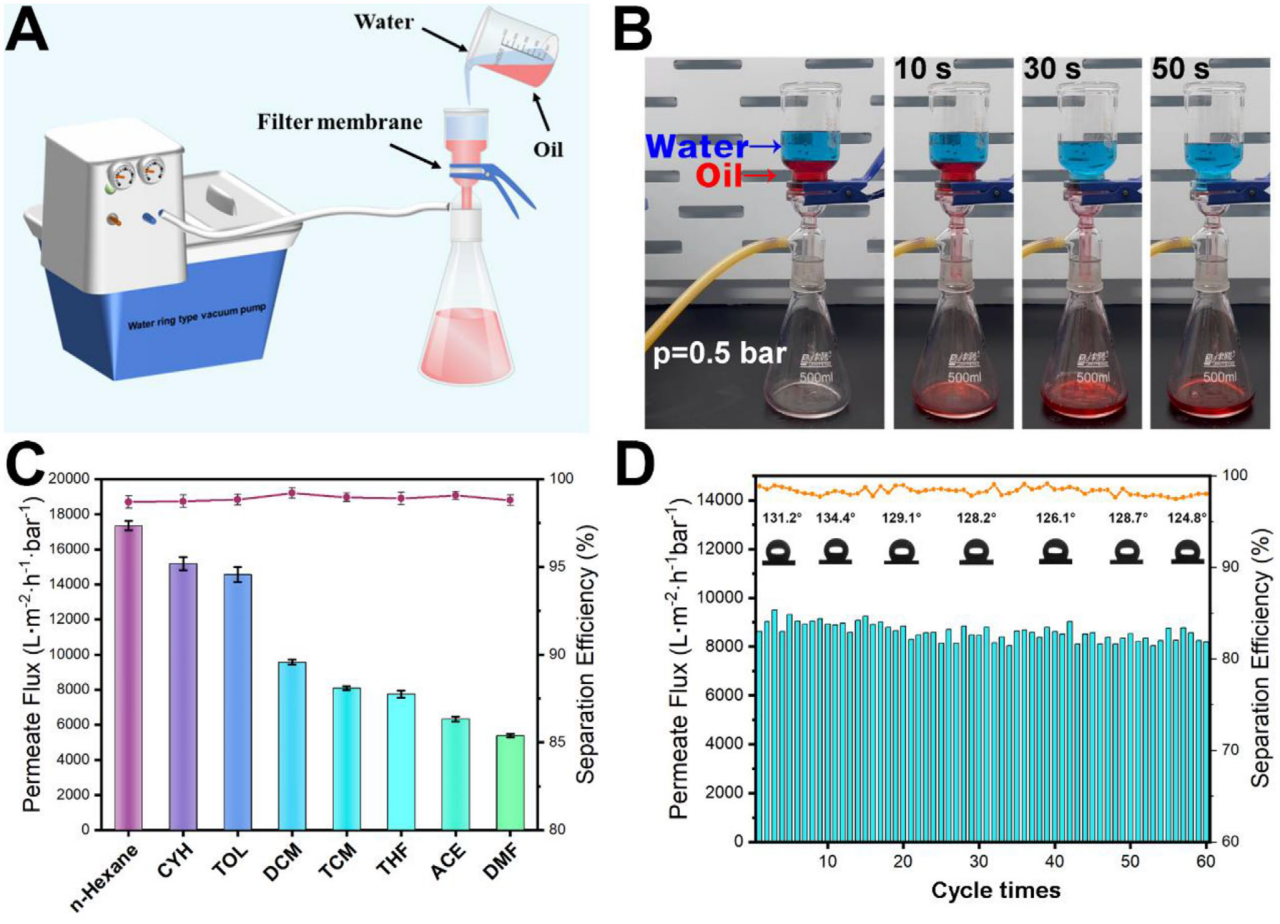
A) Schematic diagram of the oil‐water separation device, B) experimental images of the oil‐water mixture at different separation stages, C) separation efficiency (filled symbols) and permeate flux (colored bars) for various organic solvents, and D) oil‐water separation cycle test for dimethylene chloride.

CuBTCFFM is a porous membrane with a pore size of ≈1 µm and exhibits high hydrophobicity and superlipophilicity. The separation of organic solvents from oil‐water mixtures using the membrane was tested under vacuum. A mixture of 50 mL dimethylene chloride (colored with Sudan Red III) and 50 mL water (colored with Malachite Green) was prepared, which was filtrated through the hydrophobic membrane fixed in a sand core filter, as shown in **Figure** **6**A. Since dimethylene chloride is denser than water, it is located at the lower layer. When the vacuum pump is switched on, the water is repelled due to the excellent hydrophobic/lipophilic properties of the membrane (Movie S5, Supporting Information). The dimethylene chloride, on the other hand, penetrates through the membrane into the Erlenmeyer flask. Finally, all the dimethylene chloride was collected in an Erlenmeyer flask, leaving clean water in the filter cup (Figure 6B). Separation efficiency *η* and permeate flux J were used as two parameters to assess the separation performance of oily/organic solvent from water. The separation efficiency is calculated according to Equation (1):^[^ ^34^ ^]^

(1) $$\eta =\frac{m_{1}}{m_{2}}\;\times 100\%$$

Where *η* is the separation efficiency, *m*_1_ and *m*_2_ are the mass of the oil collected after separation, and the mass of oils in the oil‐water mixture before separation, respectively.

The permeate flux *J* can be calculated by Equation (2):

(2) $$J=V/\left(pA\Delta t\right)$$

Here, *V* stands for the permeated volume (L) at specific time, *p* for the test pressure (bar), *Δt* for the test time (h) and *A* for the test membrane area (m^2^).

The permeate flux and separation efficiency of various common organic solvents, including n‐hexane, cyclohexane (CYH), toluene (TOL), dimethylene chloride (DCM), trichloroethane (TCM), tetrahydrofuran (THF), acetone (ACE), and N, N‐dimethylformamide (DMF), mixed with water were measured. As illustrated in Figure 6C, the oil‐water separation efficiency for all organic substances of CuBTCFFM is above 98%. Notably, the separation efficiency of dimethylene chloride is as high as 99.4%. However, the permeate flux of different solvents varies significantly, with n‐hexane demonstrating the highest permeate flux of 17 344.2 L·m^−2^·h^−1^·bar^−1^, and DMF exhibiting the lowest permeability of 5377.6 L·m^−2^·h^−1^·bar^−1^. Organic solvents with low polarity exhibit a greater permeate flux during filtration. This phenomenon can be attributed to the strong interaction between the polar solvent and the separation membrane, which will be discussed in detail later. we designed a novel filtration device capable of indirectly reflecting continuous flux variation (Figure S2, Supporting Information). In this setup, the standard collection bottle was replaced with a graduated bottle, and the average continuous flux variation was calculated by measuring the time required to filter a fixed volume of filtrate. The results indicate that under the same operating pressure, the average continuous flux variation remains relatively stable during the initial stage. Nevertheless, as the filtrate is nearly depleted, the average continuous flux decreases sharply due to the increasing resistance from the formed filter cake layer. This observation aligns with the known permeate flux attenuation behavior characteristic of dead‐end filtration.^[^ ^35^ ^]^ Our work also demonstrates the obvious effect of the membrane thickness on the permeate flux, which is not considered in the Equation (2) when calculating permeate flux. This will be discussed in detail later.

To examine the durability of the separation membrane, the oil‐water separation performance of the dichloromethane‐water mixture after different cycles was investigated. As shown in Figure 6D, after 60 cycles, the separation efficiency of CuBTCFFM still exceeded 95%, and the change in permeate flux of the sample is negligible. At the end of each cycle, the membrane was washed with acetone and dried for WCA determination. The data indicated that after 60 cycles, there was no notable reduction in WCA (124.8°), indicating that the membrane retained its hydrophobic character. These results substantiate the notable durability of the membrane.

The effects of water‐oil ratio of high internal phase emulsion, and MOFs addition on the microstructure and separation performance of the membranes were further investigated. To ensure stability and prevent delamination of the emulsions, a maximum fluorine addition of 10% was selected for membrane preparation. SEM images (Figure S3, Supporting Information) illustrate that the percentage of water phase has an impact on the pore size and integrity of the membrane. The results of the oil‐water separation experiments indicate that the membrane achieves the highest separation efficiency at a water phase percentage of 78% (Figure S4, Supporting Information). Although the permeate flux is greatest at 85% water content, SEM images demonstrate that the pores of membrane have been destroyed and that more water molecules will pass through, which will impair the separation efficiency. CuBTCFFMs with varying amounts of CuBTC were prepared and confirmed through ATR‐FTIR (Figure S5, Supporting Information). However, excessive addition of CuBTC can lead to pore clogging and the formation of a closed pore structure (Figure S6, Supporting Information). The experiments also show that CuBTCFFM has the best separation efficiency when the CuBTC doping amount is 20% (Figure S7, Supporting Information).

**Figure 7:**
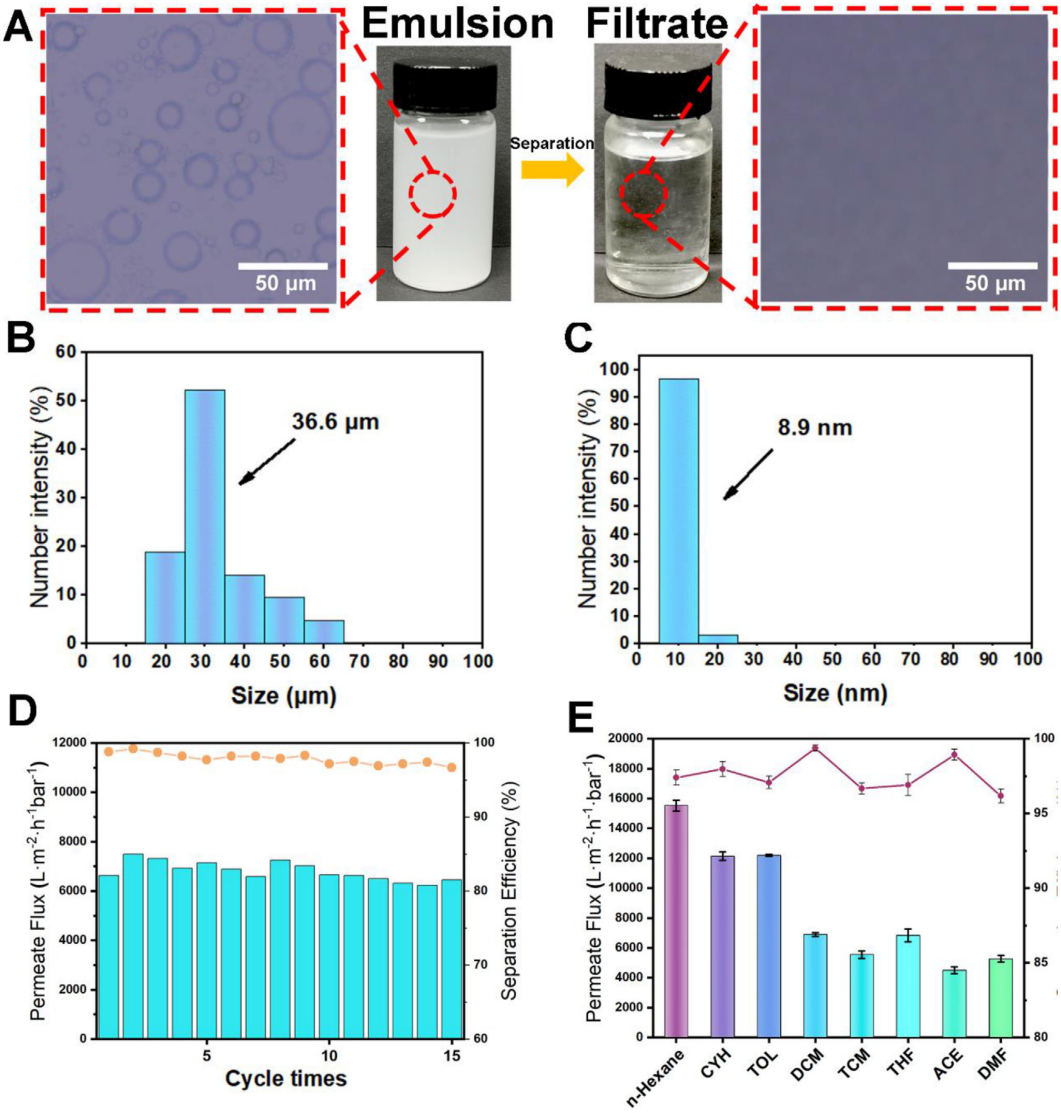
A) Photos of the water‐in‐dimethylene chloride emulsion before and after separation and corresponding optical microscopic images, particle size distribution of the emulsion (B) before and (C) after separation of W/O emulsions, D) permeate flux and separation efficiency of CuBTCFFM for the continuous separation, E) separation efficiency and permeate flux for various W/O emulsions.

The treatment of emulsified oil−water mixtures is particularly critical yet challenging, due to wastewater discharge in many industrial processes. As shown in **Figure** **7**A, the aqueous dimethylene chloride emulsion appeared a milky white liquid before separation. Microscopic images showed that the emulsion contained droplets of various sizes, and some were in the micrometer size range (Figure 7B). After separation using CuBTCFFM, only droplets smaller than 10 nm were observed (Figure 7C), demonstrating that CuBTCFFM is capable of separating not only oil‐water mixtures but also emulsions. It exhibited a remarkable separation efficiency of 99.4% and a high permeate flux of 6990.6 L·m^−2^·h^−1^·bar^−1^ (Figure 7D). The permeate flux and separation efficiency of various common organic solvents forming water‐in‐oil emulsions were determined. As shown in Figure 7E, the separation efficiency of CuBTCFFM for all organic emulsions was above 96%. The water‐in‐oil emulsions formed by solvents with smaller polarity also had a relatively high permeate flux (15 526.6 L·m^−2^·h^−1^·bar^−1^). More importantly, after 15 separation cycles, the membrane maintained the high separation efficiency and high permeate flux. By comparing and analyzing the XRD patterns of CuBTC‐FFM before and after oil‐water separation (Figure S8, Supporting Information), as well as examining the changes in SEM‐EDS results on both the membrane surface and interior(Figure S9, Supporting Information), it was found that CuBTC‐FFM can maintain structural integrity even after prolonged oil‐water separation operations. This demonstrates its excellent durability in emulsion separation.

We initially aimed to compare the results of this study with data reported in the literature. However, the membranes reported in the literature exhibit varying thicknesses, and it is evident that separation performance is influenced by membrane thickness. To investigate this hypothesis, we fabricated membranes with thicknesses ranging from 136 to 1290 µm and tested the oil‐water separation performance. We found that when the thickness of CuBTCFFM is 136 µm, the permeate flux of n‐hexane is 60 854.3 L·m^−2^·h^−1^·bar^−1^ and the separation efficiency is 99.0%. Compared with thick CuBTCFFM, the permeate flux is increased by 250.8% in thin CuBTCFFM. (Table S2, Supporting Information). As the membrane thickness increased, separation efficiency remained relatively stable, but significant declines in permeate flux were observed for various solvents, including n‐hexane, toluene and dimethylene chloride (Figure S10, Supporting Information). When the thickness exceeded a threshold (e.g., 1030 µm in our case), the permeate flux decreased sharply, likely due to insufficient driving pressure to force liquid through the membrane. By fitting the permeate flux and thickness data up to 975 µm, an exponential relationship with three parameters was derived, Equation (3).

(3) $$J=Ae^{-T/B}+C\;$$

Where *J* and *T* are the permeate flux and membrane thickness. *A*, *B*, and *C* are the fitting parameters. To validate the universality of this exponential relationship across different membrane systems, commercially available hydrophilic membranes (Cellulose acetate, Polyether sulfone (PES), and Nylon membranes) by layering them to vary thickness were prepared, and the water permeate flux of these membranes with different thickness were measured. Fitting the data revealed a similar exponential relationship. The parameters of the exponential equation differed for each liquid and each membrane system, suggesting that these parameters are intrinsic to the specific properties of the membranes and the separated liquids.

**Figure 8:**
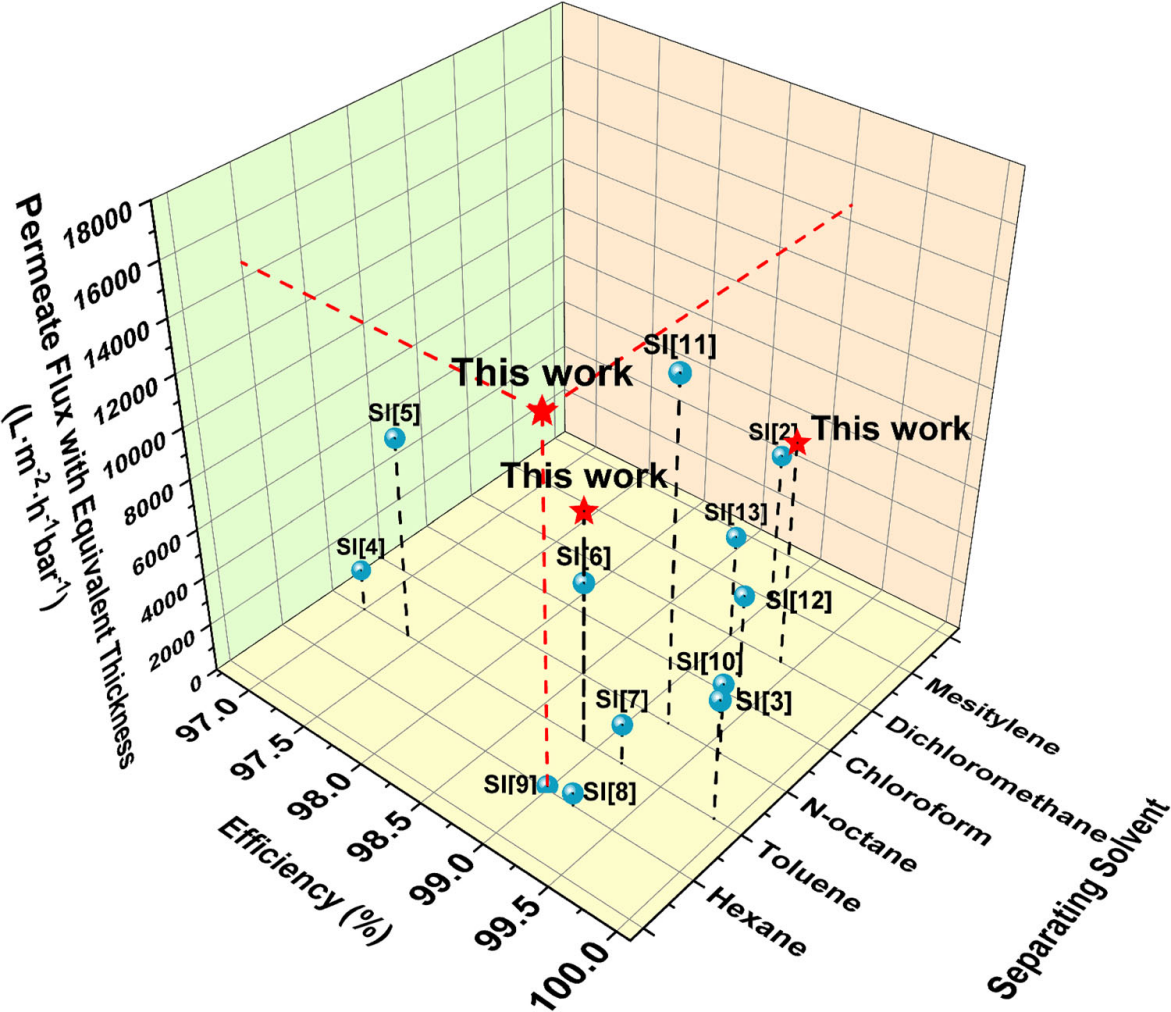
Comparison of separation efficiency and permeate flux with the equivalent thickness reported in previous works. Th permeate flux with the equivalent thickness is calculated based on the fitted equations in Figure S9 (Supporting Information).

Based on this exponential relationship, we extrapolated the permeate flux data from this study to the specific thickness reported in the literature, enabling the direct comparison of permeate flux data across membranes with equivalent thickness. CuBTCFFM demonstrates significant potential as a mixed‐matrix membrane for oil‐water separation (**Figure** **8**; Table S2, Supporting Information). It was observed that the enhancement of hexane permeate flux for the equivalent thickness was approximately 103 times higher than that of other membranes, representing a significant improvement compared to previous research.^[^ ^36^ ^]^ The permeate flux of dimethylene chloride^[^ ^37^ ^]^ and toluene^[^ ^38^ ^]^ exhibited an increase of 390% and 136%, respectively. Furthermore, in comparison with previous studies, our research also indicates that despite the greater thickness of our membrane, it still achieves a high level of membrane permeate flux, demonstrating that our membrane has the advantage of being able to withstand higher water pressure.

### 2.3 Durability of CuBTCFFM: Chemical Stability and Mechanical Integrity

#### 2.3.1 Chemical Stability

**Figure 9:**
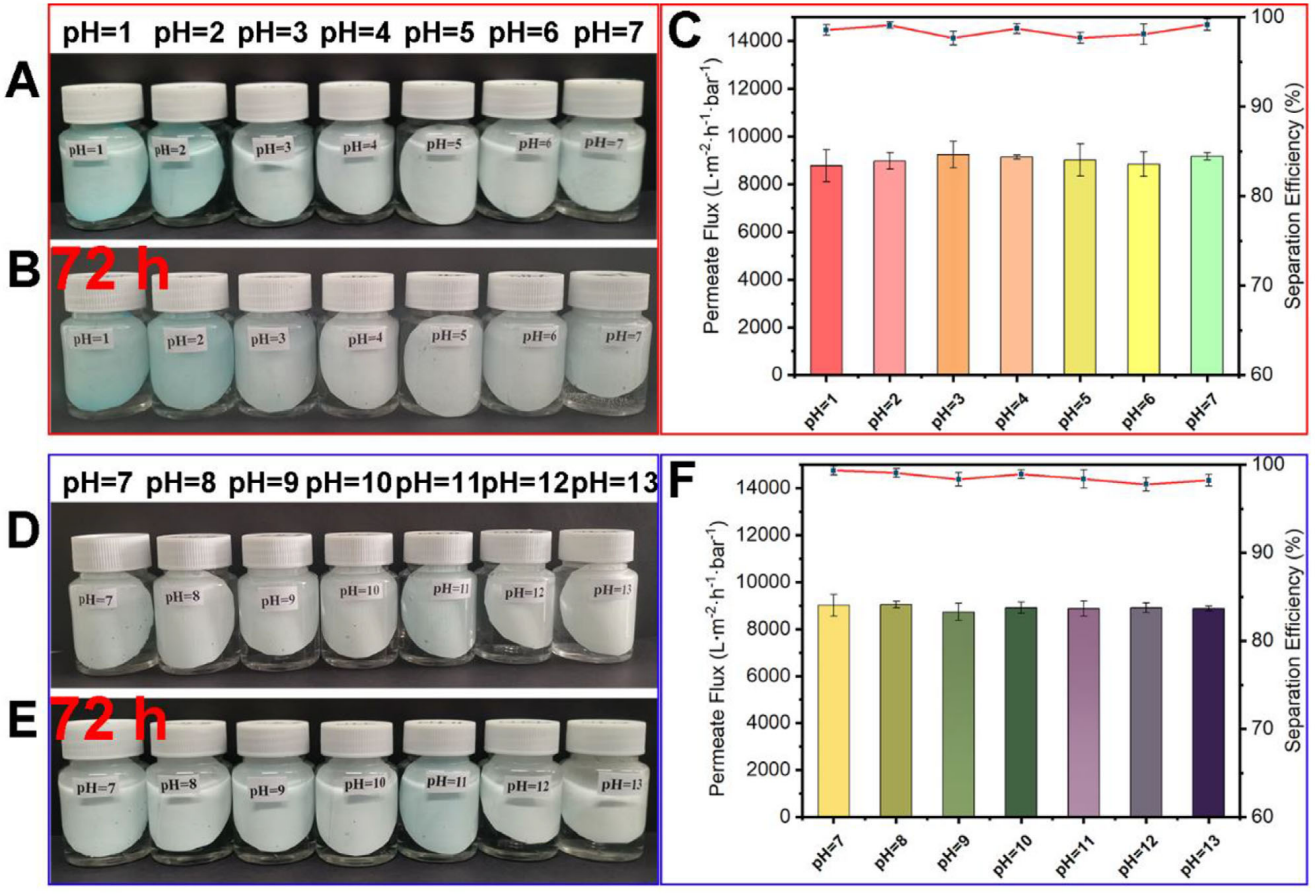
Chemical stability tests of CuBTCFFM: Immersing in (A and B) acidic and (D and E) basic aqueous solutions with different pH values for 72 h. Permeate flux and separation efficiency of the membrane after immersion in (C) acidic and (F) basic solution for 72 h.

Properties featuring enhanced chemical stability and mechanical integrity play a pivotal role in the practical applications of intricate oil‐water separation. To evaluate the separation efficiency of CuBTCFFM under acidic and basic conditions, the membrane was soaked in acidic solutions (**Figure** **9**A,B) and basic solutions (Figure 9D,E) with different pH values for 72 h, followed by testing the contact angle and oil‐water separation performance. The results (Figure 9C,F) show that CuBTCFFM exhibits excellent resistance to acid and base. No significant deterioration observed in the appearance, and the permeate flux and separation efficiency are remained stable, although. These findings demonstrate that CuBTCFFM possesses outstanding an excellent chemical corrosion resistance.

#### 2.3.2 Water Resistance

**Figure 10:**
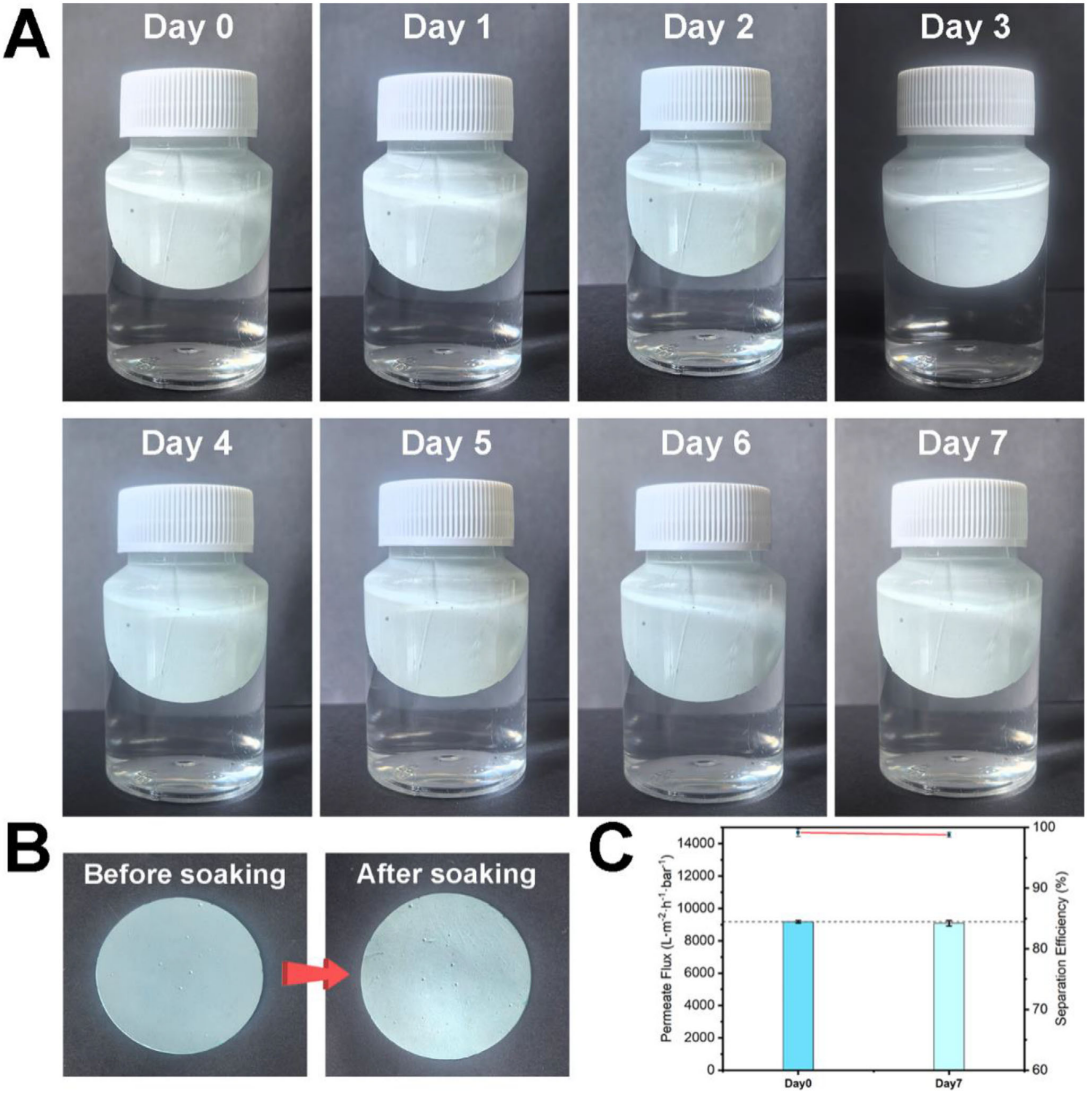
Water resistance tests of CuBTCFFM: A) The entire process of membrane soaking for 7 days, and B) states before and after soaking. C) Permeate flux and separation efficiency of the membrane before and after soaking in water for 7 days.

It is well established that CuBTC exhibits poor stability in aqueous environments over prolonged periods. In this study, CuBTC was encapsulated within the membrane matrix through high internal phase emulsion polymerization. The incorporation of fluorine‐containing functional groups further improved the hydrophobicity of the material. To evaluate the water resistance of CuBTC within the CuBTCFFM membrane, a 7‐day water soaking test was conducted (**Figure** **10**A,B). The membrane flux and separation efficiency were measured before and after soaking (Figure 10C). The results demonstrated minimal changes in both flux and separation performance, indicating that the membrane retained its effectiveness in oil‐water separation. These findings confirm that CuBTCFFM exhibits excellent water resistance.

#### 2.3.3 Organic Solvents Resistance

**Figure 11:**
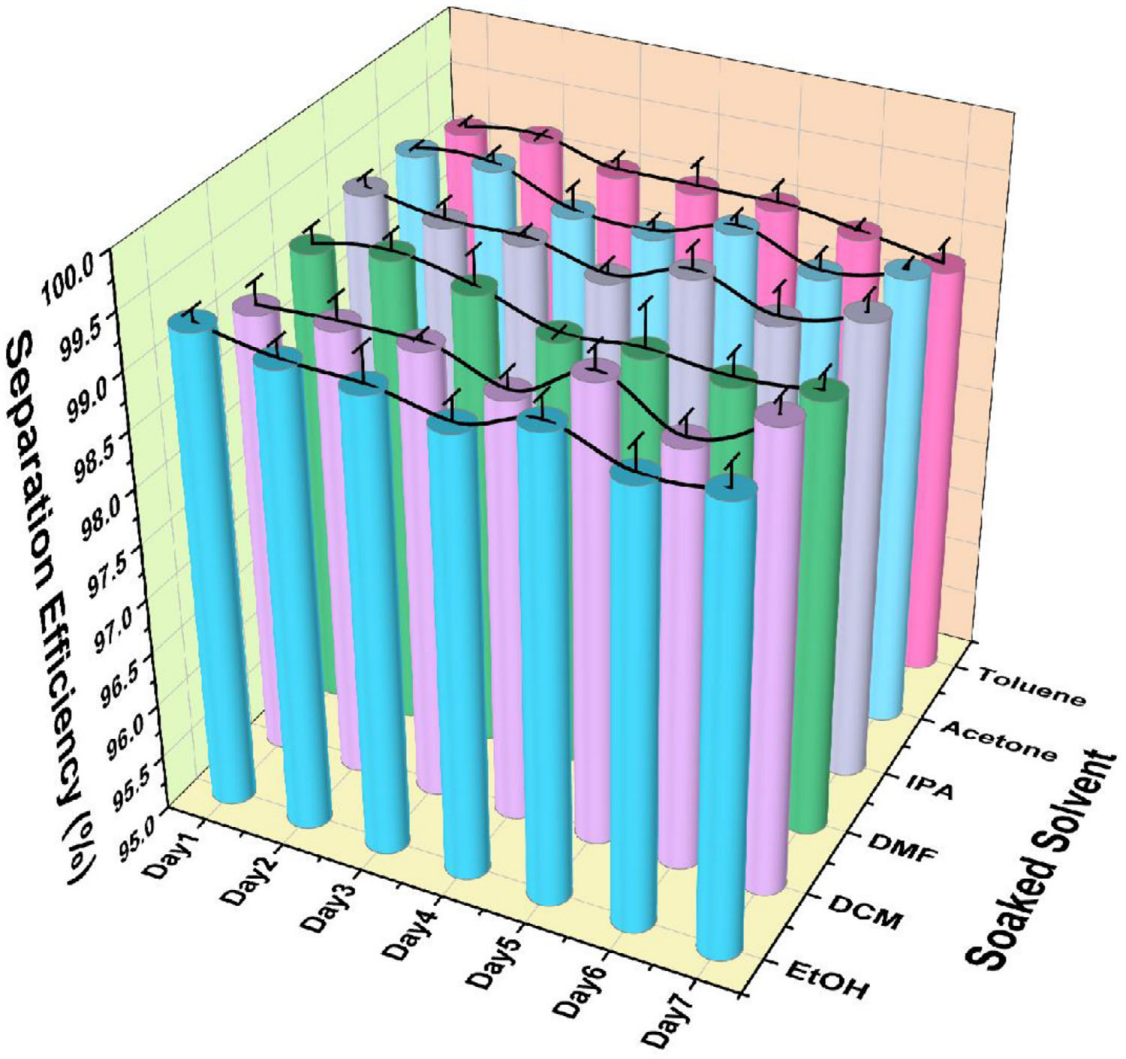
Performance tests of CuBTCFFM after soaked in various solvents for different days.

Long‐term immersion experiments were conducted to study the stability of CuBTCFFM in various solvents, including ethanol (EtOH), dimethylene chloride (DCM), dimethylformamide (DMF), isopropanol (IPA), acetone and toluene at 20 °C. CuBTCFFM was immersed in each solvent for 7 days. As shown in **Figure** **11**, the separation efficiency of CuBTCFFM for dimethylene chloride showed no significant change with increasing immersion time, consistently remaining above 98%. These results demonstrate that the prepared membranes exhibit excellent resistance to various solvent exposures.

#### 2.3.4 Abrasion Resistance

**Figure 12:**
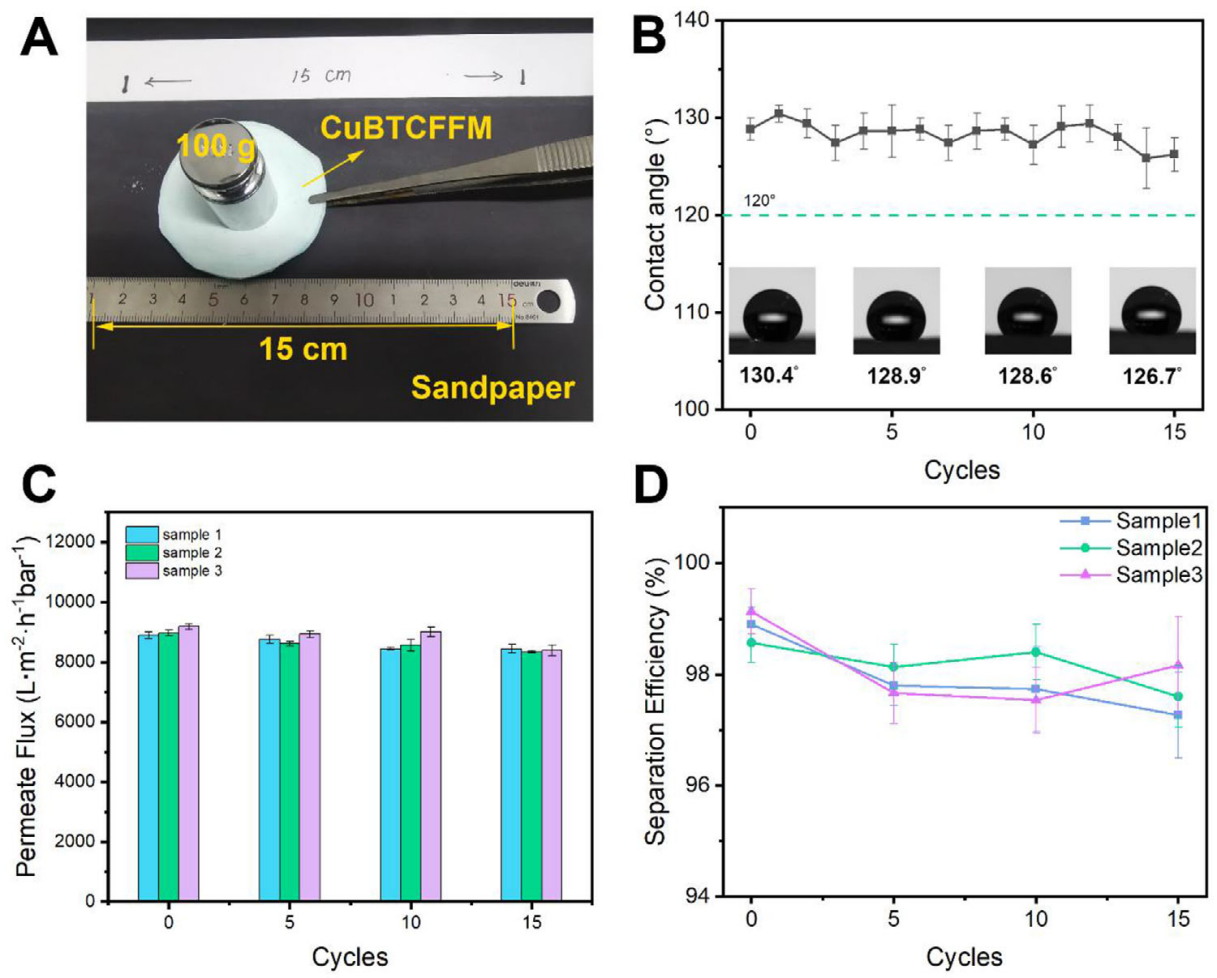
A) Schematic diagram of the sandpaper wear experiment, B) changes in WCA on the surface of CuBTCFFM‐20% during the wear cycle, C) Permeate flux, and D) separation efficiency of the samples after different cyclic abrasion.

To further evaluate the separation efficiency of CuBTCFFM in harsh environments, membrane was subjected to wear treatment. The CuBTCFFM was placed on 200 mesh sandpaper, and a weight of 100 g was applied as it was dragged 15 cm across the sandpaper (**Figure** **12**A). This process, repeated 15 times, simulated a single cycle of abrasion. As shown in Figure 12B, the repeated friction has minimal effect on both the permeate flux, separation efficiency and contact angle of the samples (Figure 12C,D). The slight increase in surface roughness due to friction resulted in a marginal decrease in the contact angle, while good hydrophobicity is maintained. These results demonstrate the excellent wear resistance of CuBTCFFM under 780 Pa.

### 2.4 Oil‐Water Separation Mechanism of CuBTCFFM

A comprehensive review and analysis on the separation mechanisms of MOF‐containing hydrophobic materials for oil‐water separation have been conducted (**Table** **1**), which primarily includes the mechanism based on size sieving effect, hydrophilic‐hydrophobic surface wettability, electrostatic interactions, and capillary forces. To determine which factors dominate among the aforementioned mechanism, we conducted the following experimental and simulation studies.

**Table 1:** Separation mechanism of oil‐water separation by different hydrophobic membranes containing MOFs.

Materials	Matrix	MOFs	WCA	Separation mechanism	References
DTMS@HKUST‐1 wood membrane	Wood sponge	HKUST‐1	162.9°	Surface wettability, size sieving effect	[39]
SA‐HKUST‐1‐CM	Copper mesh	HKUST‐1	154.6°	Surface wettability, capillary force‐based separation	[40]
CuBTC/Cu(OH)2 NWM‐PDMS	Copper mesh	CuBTC	156.2°	Surface wettability	[41]
PAN/ZIF‐8/CNT	PAN	ZIF‐8	130°	Surface wettability, size sieving effect	[42]
CF&ZIF‐8&PDMS	Cotton fabric	ZIF‐8	155°	Surface wettability	[43]
H‐Zn/Zr MOF‐NF@PDMS	Cotton fabric	Zn/Zr MOF	161°	Surface wettability	[44]
2CF_3_‐UiO‐66@CT	Cotton fabric	UiO‐66	164.7°	Surface wettability	[45]
D6/TiO_2_/MoS_2_/NiCo‐NC/PVDF	PVDF	NiCo‐MOF	162°	Surface wettability, electrostatic interaction	[46]

Two control membranes were prepared using the same fabrication method: one containing only TFEMA (FFM) and the other containing only MOFs (CuBTCEFM). SEM analysis revealed that the pore sizes of these two control membranes were nearly identical to that of CuBTCFFM (Figure S11, Supporting Information). However, it was observed that water molecules can freely permeate through these membranes indicates that none of them exhibit effective oil‐water separation performance. This suggests that the mechanism underlying water droplet removal from emulsions does not rely on the size sieving effect. Based on this phenomenon, it can be inferred that the hydrophilic‐hydrophobic wetting mechanism may be involved. According to Laplace's law, when the water contact angle (WCA) exceeds 90°, the intrusion pressure (ΔP) becomes positive, enabling CuBTCFFM to withstand a certain level of pressure and prevent water from penetrating its surface. The higher the WCA, the greater the ΔP. Although CuBTCFFM is not classified as a superhydrophobic membrane, it demonstrates the ability to endure considerable operating pressure (>1 bar), surpassing many superhydrophobic membranes. This implies that oil‐water separation is not solely governed by the hydrophilic‐hydrophobic wetting mechanism. Furthermore, control membranes, with several commonly used MOFs—MIL‐101(Fe), ZIF‐8, and UiO‐66, were fabricated using the HIPE template method (Figure S12, Supporting Information). Unfortunately, although these membrane materials exhibit hydrophobic characteristics (90° < WCA < 150°), they do not demonstrate effective oil‐water separation performance. In other words, the combination of CuBTC with TFEMA generates enhanced water repellency, which improved separation efficiency.

**Figure 13:**
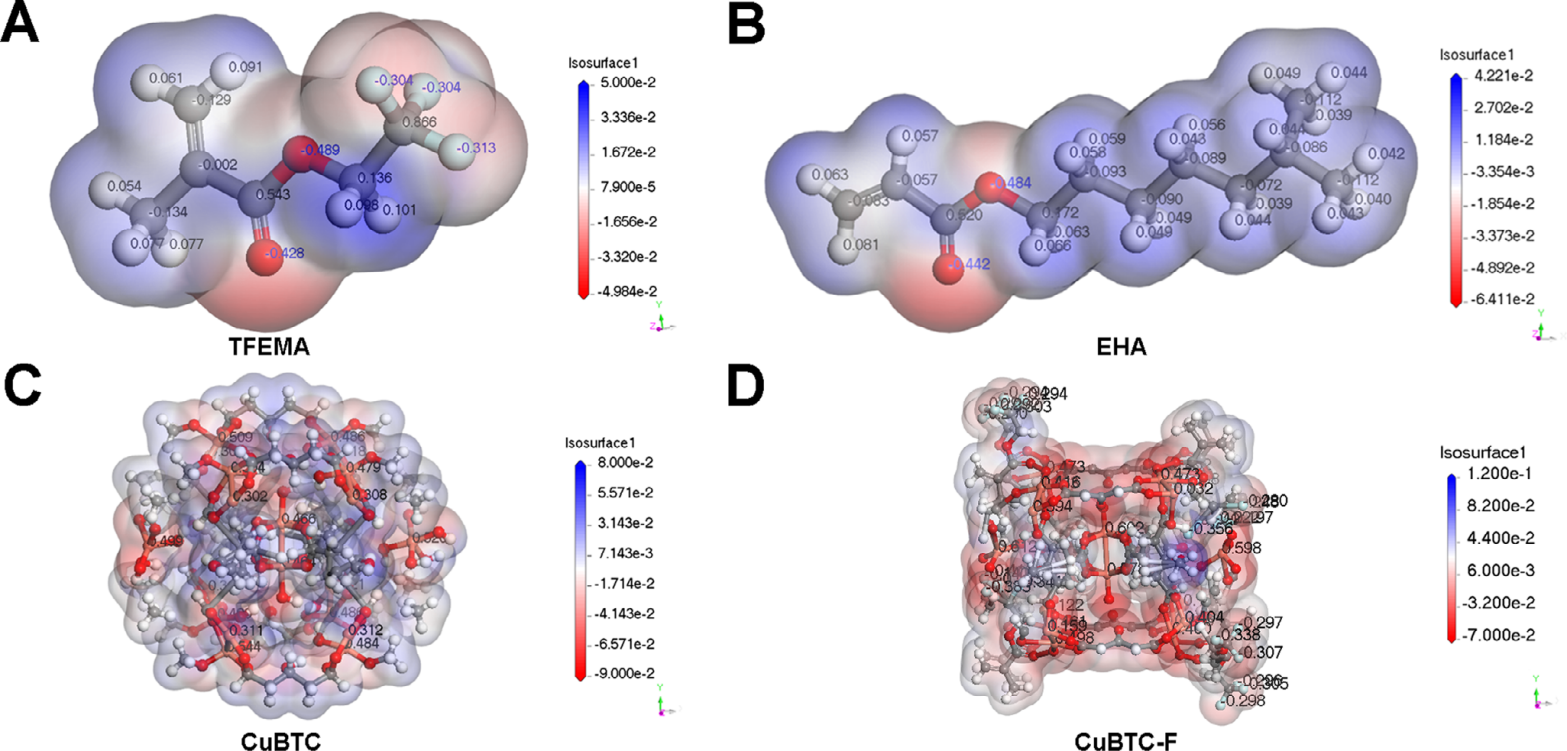
Electrostatic potential of A) TFEMA, B) EHA, C) CuBTC, and D) CuBTC‐TFEMA (CuBTC‐F) complex via hydrogen bonding.

The electrostatic interactions of CuBTC with monomers in the system were simulated. **Figure** **13**A–D displays the electrostatic potential of TFEMA, EHA, CuBTC, and CuBTC‐TFEMA (CuBTC‐F) complex, respectively. The electrostatic potential reflects the potential distribution generated by the molecular charge. For TFEMA, the negatively charged regions are predominantly located near the oxygen atoms of the ester group and around the C─F bond (Figure 13A). For EHA, only the oxygen atoms of the ester group are negatively charged (Figure 13B). The outermost O─H bonds of CuBTC molecule exhibit positive charges (Figure 13C). The C─F bonds and ester groups in TFEMA act as the principal hydrogen bond acceptors, while the positively charged O─H bonds on the surface of CuBTC function as the primary hydrogen bond donors. Thus, the highest electrostatic sites of CuBTC were attracted to highly negative regions of TFEMA (Figure 13D), predicted using the Fukui function of DMol3.^[^ ^47^ ^]^ Subsequently, the double bonds of TFEMA undergo photoinitiated polymerization, resulting in TFEMA being firmly encapsulated on the outer surface of CuBTC. As a result, the surface of CuBTC becomes surrounded by F atoms, changing the surface of CuBTC being negatively charged. The negatively charged CuBTC enhances the interaction with water molecules and thus obstruct the passage of water molecules, which is further verified using the molecular dynamic simulation below.

**Figure 14:**
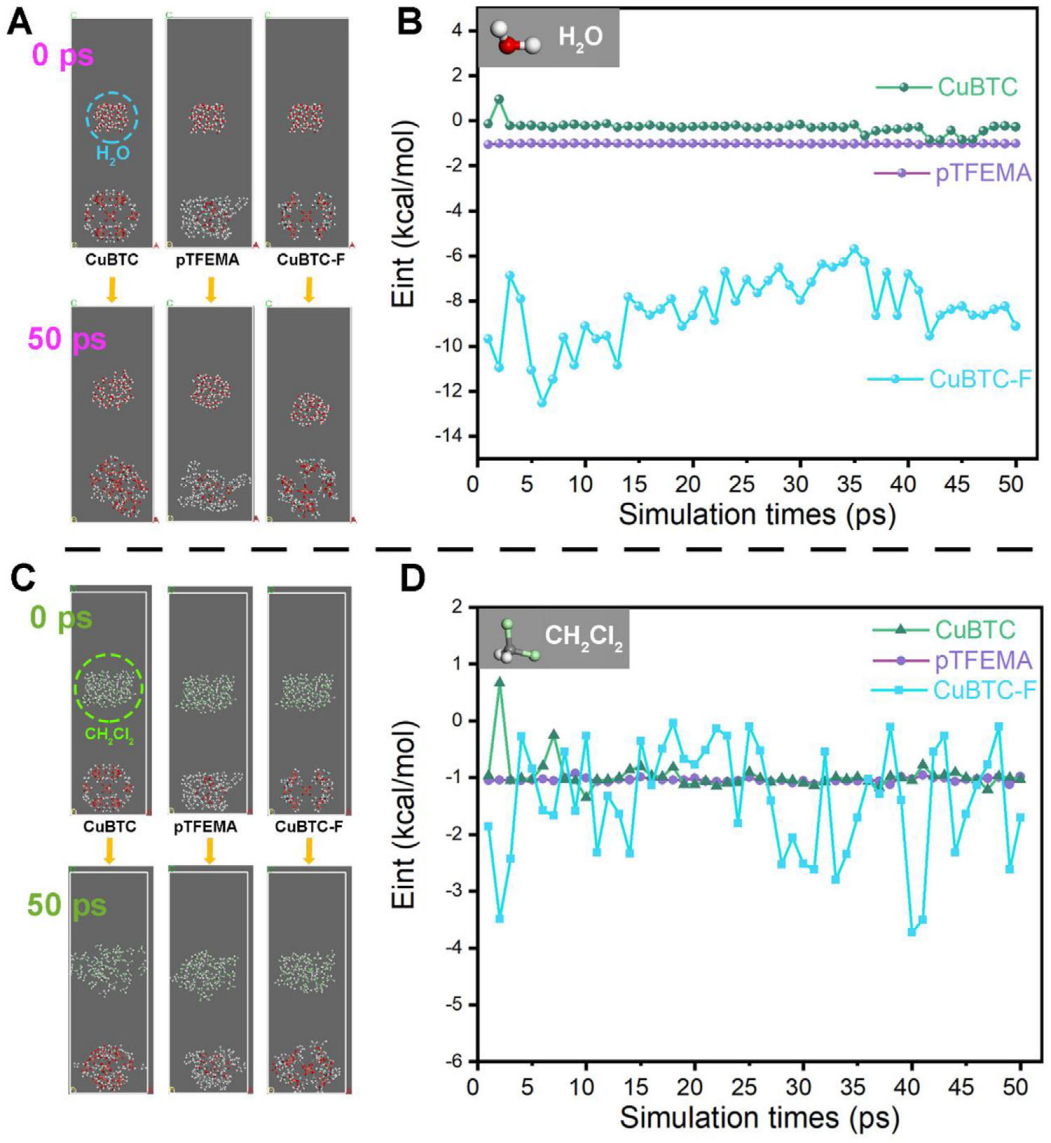
Interaction energy simulation of CuBTC, fluorinated polymer (pTFEMA) and fluorinated polymer CuBTC complex (CuBTC‐F) with (A and B) water molecules and with (C and D) dimethylene chloride molecules.

Interaction forces arise when particles come close to each other or to a surface, encompassing van der Waals forces, hydrogen bonding and electrostatic interactions. These forces reflect the magnitude of interactions between a material surface and surrounding molecules. Forcite module was used to simulate the interaction energies of CuBTC, fluorinated polymer (pTFEMA) and CuBTC complex (CuBTC‐F) molecules at the solid/water (**Figure** **14**A) or solid/dimethylene chloride (Figure 14C) interfaces.^[^ ^48^ ^]^ The interaction energy (*E*_int_) was calculated using Equation (4):

(4) $$E_{\mathrm{int}}=E_{\mathrm{Total}}\;-\left(E_{\mathrm{Label}}+E_{\mathrm{Sol}}\right)$$

*E*_Total_ represents the total interaction energy of two phases. *E*_Label_ refers to the interaction energy within the solid phase, while *E*_Sol_ indicates the interaction energy within the liquid phase.

The interaction energy between CuBTC and water after 50 ps is approximately ‐0.302 kcal·mol^−1^ (Figure 14B), indicating a weak attraction, which aligns with the hydrophilic nature of CuBTC. The interaction energy between the fluoropolymer pTFEMA and water is approximately ‐1.02 kcal·mol^−1^ (Figure 14B), attributed to the polar hydrophobicity arising from the electrostatic interaction between C─F bonds and water molecules.

When CuBTC‐F, a molecule complex containing fluorine monomers, is introduced onto the surface, an electrostatic field is generated between MOFs and C─F bonds, resulting in a strong electrostatic polar force. The strong electronegativity of the fluorine atoms enhances the interaction force with the water molecules on the surface, resulting in a binding force of ≈‐8.34 kcal·mol^−1^ (Figure 14B). Such strong interactions hinder the movement of water molecules, making it difficult for them to escape and pass through the surface. As a result, this phenomenon obstructs the passage of water molecules during oil‐water separation, ultimately leading to the effective separation. Figure 14D illustrates that the interaction energies between the three solid phases and dimethylene chloride do not exhibit a significant superposition effect, allowing the organic solvent to pass without obstruction.

**Figure 15:**
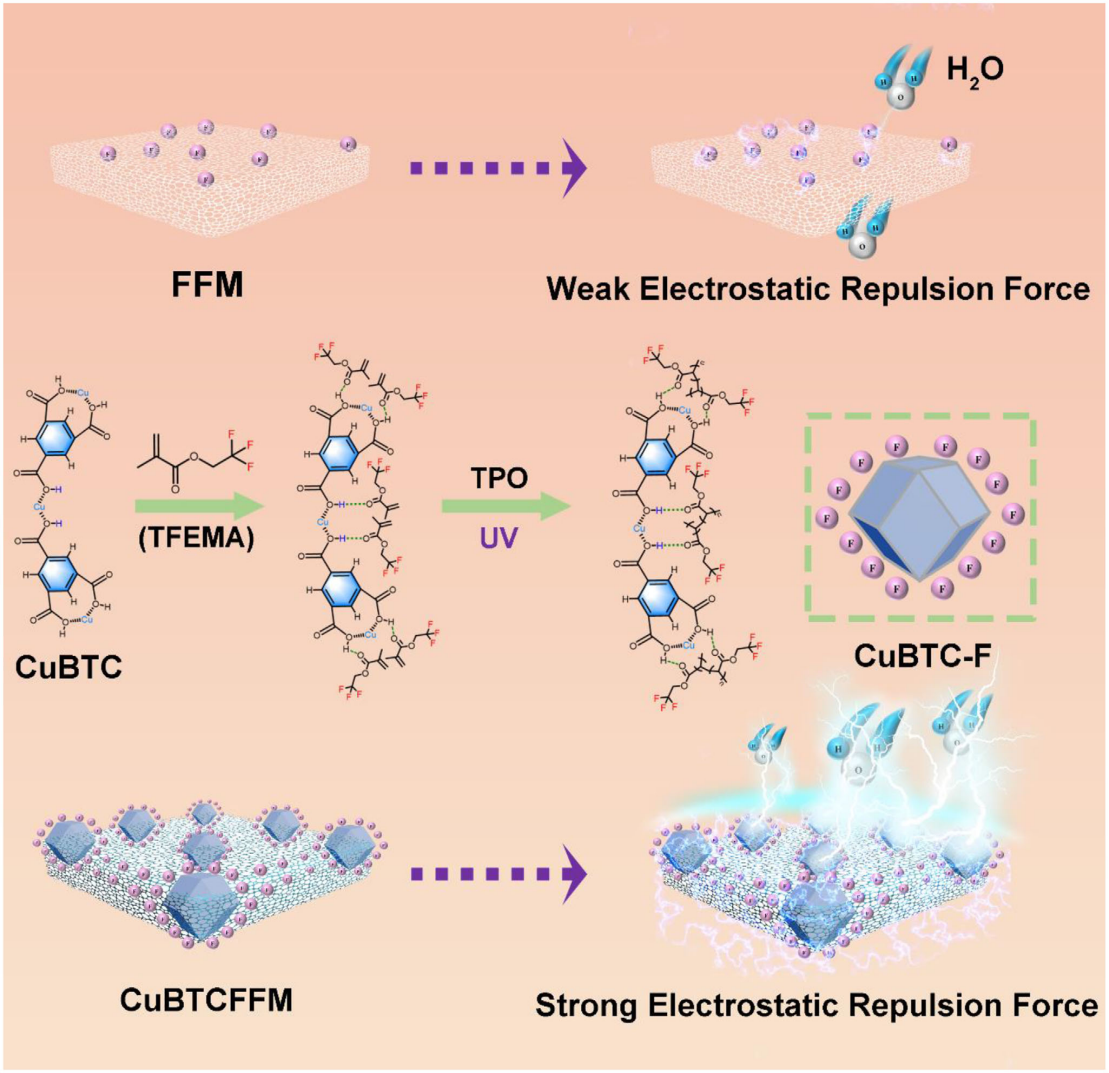
Schematic formation of fluorinated polymer CuBTC complex (CuBTC‐F) and sufficient electrostatic repulsion force toward water of CuBTCFFM covering micro‐scale pore structures.

Based on the foregoing review and analysis of diverse separation mechanisms presented in the literature, along with the outcomes of our control experiments and simulation results, we conclude that while the membrane material exhibits surface hydrophobicity capable of generating aqueous repulsion forces, this hydrophobic mechanism proves inadequate in effectively sustaining the high‐performance separation demonstrated in this study. Instead, the electrostatic repulsion force arising from the combination of CuBTC and TFEMA exerts a more pronounced influence on water molecules. The hydrogen bonds between CuBTC and TFEMA make CuBTC surrounded by C─F bonds after polymerization (**Figure** **15**). This configuration establishes a robust polar electrostatic force between the MOFs and C─F bonds, significantly increasing the electrostatic repulsion force (Figure 14B). As a result, the membrane surface exhibits the polar hydrophobicity, creating a strong barrier for polar molecules like water, which require additional energy to overcome. This strong electrostatic repulsion toward water prevents water molecules going through the membrane. The separation efficiency is ensured by this strong electrostatic repulsion force. This is also the reason that organic solvents with varying polarities exhibit different membrane permeate fluxes (recall Figure 6C). For the FFM, the electrostatic repulsion toward water is weak (Figure 14B) and water molecules can go through the membrane easily. ③Enhanced surface lipophilicity and capillary effect: Isooctyl is a typical long‐chain saturated alkyl group, which belongs to a strong lipophilic group. It cannot form hydrogen bond or dipole interaction with polar water molecules, but is easy to combine with oil phase (nonpolar/weakly polar organic matter) through van der Waals dispersion force. The OCA is close to 0, Δ*P*<0, and oil can pass through the membrane. In addition, the pore size of CuBTCFFM is about 1 µm. In the separation process, the capillary force drives the oil to enter the interior through the porous membrane, while the water remains outside due to the electrostatic polar force.

## 3 Conclusion

In summary, we have developed a simple and rapid method for the preparation of uniformly dispersed porous MOFs/polymer mixed‐matrix membranes. By utilizing a high internal phase emulsion as a template, CuBTC was evenly distributed within the oil phase, while its hydrophobicity was enhanced through modification with fluorine‐containing functional groups. The flexible monomer was rapidly polymerized under exposure to ultraviolet light and the flexible porous MOFs mixed‐matrix membrane was produced. The fabricated flexible mixed‐matrix membrane shows high hydrophobicity as well as excellent stability and durability in harsh environments. Furthermore, owing to the electrostatic polar force, the material has excellent separation efficiency and permeate flux for oil‐water mixtures and emulsions. As a consequence, it exhibits a diverse range of applications in the treatment of emulsified wastewater.

## 4 Experimental Section

### Preparation of Copper Benzene‐1,3,5‐tricarboxylate (CuBTC) MOFs

Cu(NO_3_)_2_·3H_2_O (2.92 g) was dissolved in 30 mL of distilled water designated as liquid A in an ultrasonic bath. 1.4 g of 1,3,5‐benzenetricarboxylic acid was weighed out in another beaker and dissolved in a mixture of 15 mL of anhydrous ethanol and 15 mL of DMF, referred to as liquid B. The two liquids were mixed and placed in a Teflon‐lined reactor at 120 °C for 10 h. The resulting product was centrifuged and washed three times with anhydrous ethanol. After drying in a vacuum oven, the purified product CuBTC was obtained.

### Preparation of MOF‐Doped Fluorine‐Containing Flexible Membrane (CuBTCFFM)

The fluorine‐containing, flexible CuBTC membrane was prepared by high internal phase emulsion (HIPE) template method (Figure 1). W/O HIPE is an emulsion in which a large number of water droplets are dispersed within a continuous oil phase. The volume fraction of the aqueous phase typically exceeds 74%. As the water content in the internal phase increases, the droplets gradually transition from a spherical morphology to a polyhedral skeletal structure due to compression. Upon removal of the internal phase through drying, a porous polymer material is obtained.^[^ ^49^ ^]^ First, 0.3 g CuBTC (20% organic phase) was weighed and dispersed in 5.3 g distilled water as the aqueous phase of the high inward emulsion. 0.07 g hypermer B246 (4.6% organic phase) and added 0.6 g PUDA, 0.7 g 2‐ethylhexyl acrylate and 0.15 g trifluoroethyl methacrylate (10% organic phase) as the oil phase. Finally, 0.003 g of 2‐hydroxy‐2‐methylphenylacetone and 0.003 g of diphenyl (2,4,6‐trimethylbenzoyl) phosphine oxide were added as photoinitiators and sonicated for 5 min. The mixture was poured into a 100 mL three‐way bottle. The prepared aqueous phase with dispersed CuBTC was slowly added to the mixture with mechanical shaking at 500 rpm with one drop every 3 s. Stirring continued for 10 min to ensure uniform dispersion of the W/O HIPE.

A hollow, circular silica gel pad with a thickness of 1 µm and a diameter of 5 cm was used as a mold. The high viscosity light blue emulsion was poured into the mold. The upper and lower parts were clamped with two pieces of 10 cm × 10 cm glass plate. Polymerization was completed by ultraviolet irradiation on both sides of the glass plate with a high‐intensity UV lamp (maximum power of 250 W) for 10 min. After polymerization, unreacted monomers were washed away with acetone. The resulting material was kept in an oven at 60 °C for 4 h to obtain the light blue, porous, flexible and fluorine‐containing membrane. The ratio between water and oil phase (CuBTCFFM‐70W∼CuBTCFFM‐85 W) and the content of CuBTC (CuBTCFFM‐10%∼CuBTCFFM‐50%) to synthesize different CuBTCFFM (Table S1, Supporting Information) were adjusted. If not stated otherwise, the test composition of CuBTCFFM is CuBTCFFM‐20%. Additionally, controls (FFM) were prepared without CuBTC, utilizing 0.2 mol·L^−1^ CaCl_2_·2H_2_O rather than H_2_O. On the other hand, the membrane without TFEMA (CuBTCEFM) was also prepared to ascertain the impact of fluorine‐containing monomers on the separation efficiency of the oil‐water separation.

### Molecular Dynamics Simulation

All computational simulations were performed using the Materials Studio 2023 software. The Dmol3 module was used to calculate the electrostatic potential, and the solid‐liquid interface model was constructed with the AC module. Molecular dynamics simulations were performed using the Forcite module. The COMPASS III force field was used for all simulations.

The CuBTC model diagram (4512170) was obtained from the CCDC crystal database.^[^ ^47^ ^]^ The electrostatic potential of CuBTC was calculated using the Dmol3 module. The active sites on the surface, as well as in the microporous materials, were determined using the Fukui function algorithm. The structural formula of CuBTC after reaction with TFEMA was simulated and designated CuBTC‐F. The reaction between TFEMA and EHA was modeled to simulate the fluoropolymer molecules. Based on the reactant addition ratio, a periodic amorphous unit was constructed containing 3 TFEMA and 12 EHA molecules, which comes from the ratio of the two monomers in the experiment. Subsequently, 500 simulations of disordered polymerization cross‐linking were performed. The fluoropolymer molecular with the most stable spatial configuration was selected based on the lowest energy principle and defined as polyTFEMA (pTFEMA). It was chosen for the subsequent simulation modeling of the adsorption energy.

The solid‐liquid interface model was constructed using the AC module to calculate the initial distribution of adsorption energy between molecules and water.^[^ ^48^ ^]^ A monomer system with a total density of 1 g cm^−3^ filled a cubic box with periodic boundary conditions, measuring 20 × 20 × 84 Å. The model included a polymer layer and an aqueous‐phase layer, separated by a vacuum layer with a thickness of 30 Å. The polymer layer comprises CuBTC, pTFEMA, and CuBTC‐F molecules, while the aqueous‐phase layer contains 50 randomly placed water molecules. The total number of atoms in the three polymer layers remained constant for comparison. Before modeling, the molecules were assigned force fields and charges using the Forcite module, and the molecular model's geometry was optimized through Geometry Optimization. The model with the lowest energy was used for dynamic simulations. Molecular dynamics calculations were conducted using an isothermal and isocapacitive (NVT) system at a temperature of 298 K. The polymer molecular layer was fixed at the bottom, and the molecular force field caused the water molecules to move, resulting in contact between the two phases. The simulation lasted for 50 ps with a time step of 1.0 fs. After the calculation was complete, the interaction energy change values for each polymer layer with water were output.

The method for calculating the interaction energy of the three molecules with methylene chloride is similar to the one described earlier except that 50 molecules of methylene chloride are used instead of water.

## Conflict of Interest

The authors declare no conflict of interest.

## Supporting information



Supporting Information

Supplemental Movie 1

Supplemental Movie 2

Supplemental Movie 3

Supplemental Movie 4

Supplemental Movie 5

## Data Availability

The data that support the findings of this study are available from the corresponding author upon reasonable request.
