# Supplementary material for: Freestanding CuBTC‐Doped Fluorine‐Containing Flexible Membrane with Ultrafast Oil‐Water Separation via Enhanced Electrostatic Repulsion Force
Source: Adv Sci (Weinh). 2025 Sep 14;12(45):e09657. doi: 10.1002/advs.202509657 (PMC12677696; doi:10.1002/advs.202509657)
Supplement: Supplementary file 1 — Supporting Information [file ADVS-12-e09657-s002.docx]

**Supporting Information**

**Freestanding CuBTC-doped Fluorine-containing Flexible Membrane with Ultrafast Oil-Water Separation via Enhanced Electrostatic Repulsion Force** Xiaoshuang Li^a, b^, Peize Yang^a, b^, Minghui Zhu^a, b^, Zhen Zhang^a, b^, Boyu Lei^a, b^, Guanghui Cui^c *^, Mei Yan ^a, b *^, Bing Geng^a, b *^

^a^ School of Chemistry and Chemical Engineering, University of Jinan, Jinan 250022, China.

^b^ Shandong Provincial Key Laboratory of Fluorine Chemistry and Chemical Materials, University of Jinan, Jinan 250022, China.

^c^ Drilling Fluid Research Center of Mud Service Branch, Bohai Drilling Engineer Company, China National Petroleum Corporation (CNPC), Tianjin 300280, China

E-mail addresses: [cuiguanghui@cnpc.com.cn](mailto:cuiguanghui@cnpc.com.cn) (Guanghui Cui), chm_yanm@126.com (Mei Yan), chm_gengb@ujn.edu.cn (Bing Geng)

**Table of Contents:**

| Supporting Movies | |
| --- | --- |
| Movie S1 | Oil contact angle of CuBTCFFM. |
| Movie S2 | Under-oil water contact angle of CuBTCFFM. |
| Movie S3 | Under-oil water rolling video of CuBTCFFM. |
| Movie S4 | Under-oil water rolling angle of CuBTCFFM. |
| Movie S5 | Oil/water separation by vacuum suction using CuBTCFFM. |
| Supporting Tables | |
| Table S1 | HIPEs components under different formulations. |
| Table S2 | Comparison of key performance parameters of the hydrophobic membrane with previous works. |
| Supporting Figures | |
| Figure S1 | XRD patterns of CuBTC. |
| Figure S2 | (A) Measuring device for the average continuous flux variation during oil-water separation in CuBTCFFM. (B) Average continuous flux variation of CuBTCFFM. |
| Figure S3 | SEM images of CuBTCFFMs prepared with different water-oil ratios. |
| Figure S4 | Permeate flux (colored bars), separation efficiency (filled symbols) of dichloromethane solvent from water, and water contact angle of CuBTCFFMs prepared with different water-oil ratios. |
| Figure S5 | The ATR-FTIR spectra of the CuBTCFFMs doped with different CuBTC content. |
| Figure S6 | SEM images of CuBTCFFMs doped with different CuBTC contents. |
| Figure S7 | Permeate flux (colored bars), separation efficiency (filled symbols) of dichloromethane from water, and water contact angle of CuBTCFFMs doped with different CuBTC content. |
| Figure S8 | XRD patterns of CuBTCFFM before and after oil-water separation. |
| Figure S9 | SEM images and EDS analysis of CuBTCFFM (surface and cross-sectional) before and after oil-water separation. |
| Figure S10 | Permeate flux for membranes of varying thickness: (A) CuBTCFFM (The solvent is dichloromethane), (B) CuBTCFFM (The solvent is hexane) and (C) CuBTCFFM (The solvent is toluene), (D) Nylon membrane, (E) Polyether sulfone membrane (PES), (F) Cellulose acetate membrane (CA-CN). |
| Figure S11 | (A) SEM image of the surface, (B)water contact angle, and (C) interaction energy simulation (pink) of CuBTCEFM, (D) experimental images of CuBTCEFM for different separation stages of oil-water mixtures. |
| Figure S12 | SEM images and EDS analysis of MIL-101(Fe)FFM, ZIF-8FFM, and UiO-66FFM doped with different MOFs prepared by HIPE template method. |

**Supplementary Text**

**Materials**

Copper nitrate trihydrate, 1,3,5-benzenetricarboxylic acid (BTC) and 2-ethylhexyl acrylate (2-EHA) were obtained from Shanghai Aladdin Bio-Chem Technology Co, Ltd (Shanghai, China). 2,2,2-Trifluoroethyl methacrylate (TFEMA) was obtained from Harbin Saiojia Fluoro Silicon Chemical Co, LTD (Harbin, China). Hypermer B246 is a copolymer consisting of polyhydroxystearic acid and polyethylene glycol and was supplied by Croda, USA. Polyurethane diacrylate (PUDA) is an aliphatic polyurethane diacrylate oligomer diluted with 1,6-hexanediol diacrylate monomer supplied by Cytec Company, Belgium. Calcium chloride dihydrate (CaCl_2_⋅2H_2_O) was from Sigma Aldrich (USA). The dye Sudan III was supplied by Sinopharmed Chemical Reagent Co, LTD (Shanghai, China). The photoinitiator 2-hydroxy-2-methylphenylacetone/ diphenyl (2,4,6-trimethylbenzoyl) phosphine oxide (TPO) was provided by Alighting Biochemical Technology Co, LTD (Shanghai, China). Methanol (> 99.5%), ethanol (> 99.7%), n-hexane (> 97%), cyclohexane (CYH, > 99.7%), toluene (TOL, > 99.5%), dimethylene chloride (DCM, > 99.5%), trichloroethane (TCM, > 99.5%), tetrahydrofuran (THF, > 99.5%), acetone (ACE, > 99.5%), and N, N-dimethylformamide (DMF, > 99.5%) were all produced by Tianjin Fuyu Chemical Co, Ltd (CN).

**Characterization**

The structure of the material was characterized by Fourier infrared spectroscopy (Nicolet iS10, USA) with a scanning range of 4000-500 cm^-1^. Structural analysis of the samples was conducted with an X-ray diffractometer (XRD, SmartlabSE, Japan). Polarizing microscope (LV100POL, Japan) was used to analyze the micro-morphology of emulsion. Scanning electron microscope (Gemini300, Germany) was used to analyze the microscopic morphology of the surface and cross-section of CuBTCFFM. The small sample was fixed on the sample stage with conductive adhesive. It was then coated with sputtered gold (60 s, 220 V, 6 Pa). The samples for cross-sectional SEM characterization were prepared by careful snapping after immersion in liquid nitrogen for 30 s. The content and distribution of elements on the surface of CuBTCFFM were characterized by energy dispersion spectroscopy (EDS with a secondary electron mode and an accelerating voltage of 20 kV, X-MAX-50, UK). The hydrophobicity of CuBTCFFM was measured at room temperature using the fully automated optical video contact angle measuring device OCA40 (Data Physics, Germany) by testing at five different positions and then determining the average value. X-ray photoelectron spectroscopy (XPS) was investigated on the Thermo EscaLab 250Xi (USA) with an Al-Kα source (15 kV and 25 W) and a pass energy of 100 eV to analyze the surface elements of the CuBTCFFM. The thermal stability of CuBTCFFM was investigated by thermogravimetric analysis (TGA, TA Instruments SDT2960) in an argon atmosphere at a rate of 10 °C/min.

**X-ray computed tomography (X-CT)**

The three-dimensional (3D) pore structure of CuBTCFFM was characterized using a high-resolution scanner (ZEISS Xradia510, Germany). The high voltage was set to 50 keV, and the 3D spatial resolution was set to 700 nm. The acquisition time was 4.97 hours. The images were analyzed using Aviz software, Version 2020.2, which is available at https://www.avizo3d.com/. The samples were cut into cylinders with a diameter of 100 μm and a height of 700 μm using the Extract Subvolume command. To preprocess image data, extract pores, and calculate porosity, please refer to the previous methods^[1]^. Pore extraction was performed using the Interactive Thresholding command on the original slice images. It is important to note that the membrane surface is grey, the CuBTC is white, and the pores appear black. Subsequently, the black pixel blocks were extracted to calculate porosity and construct the pore network model inside the CuBTCFFM.

**Supplementary Movie, Tables and Figures**

**Movie S1.** Oil contact angle of CuBTCFFM.

**Movie S2.** Under-oil water contact angle of CuBTCFFM.

**Movie S3.** Under-oil water rolling video of CuBTCFFM.

**Movie S4.** Under-oil water rolling angle of CuBTCFFM.

**Movie S5.** Oil/water separation by vacuum suction using CuBTCFFM.

**Table S1**. HIPEs components under different formulations.

| Sample | 2-EHA  (g) | TFEMA  (g) | CuBTC  (g) | H_2_O  (g) |
| --- | --- | --- | --- | --- |
| FFM | 0.7 | 0.15 | 0 | （CaCl_2_·2H_2_O） 5.3 |
| CuBTCEFM | 0.85 | 0 | 0.15 | 5.3 |
| CuBTCFFM-70W | 0.7 | 0.15 | 0.3 | 3.55 |
| CuBTCFFM-75W | 0.7 | 0.15 | 0.3 | 4.56 |
| CuBTCFFM-76W | 0.7 | 0.15 | 0.3 | 4.81 |
| CuBTCFFM-77W | 0.7 | 0.15 | 0.3 | 5.08 |
| CuBTCFFM-78W | 0.7 | 0.15 | 0.3 | 5.3 |
| CuBTCFFM-79W | 0.7 | 0.15 | 0.3 | 5.71 |
| CuBTCFFM-80W | 0.7 | 0.15 | 0.3 | 6.08 |
| CuBTCFFM-85W | 0.7 | 0.15 | 0.3 | 8.61 |
| CuBTCFFM-10% | 0.7 | 0.15 | 0.15 | 5.3 |
| CuBTCFFM-20% | 0.7 | 0.15 | 0.3 | 5.3 |
| CuBTCFFM-30% | 0.7 | 0.15 | 0.45 | 5.3 |
| CuBTCFFM-40% | 0.7 | 0.15 | 0.6 | 5.3 |
| CuBTCFFM-50% | 0.7 | 0.15 | 0.75 | 5.3 |

**Table S2**. Comparison of key performance parameters of the hydrophobic membrane with previous works.

| Materials | Emulsion | Thickness (μm) | Driver (bar) | Separation  Efficiency | Permeate flux  (L·m^-2^·h^-1^bar^-1^) | Permeate flux of CuBTCFFM with equivalent thickness (L·m^-2^·h^-1^bar^-1^) | Refs |
| --- | --- | --- | --- | --- | --- | --- | --- |
| HNTC-FG-PU sponges | water/  mesitylene | 10000 | 0.745 | 99% | 6295.3 | - | ^[2]^ |
| PS-g-CNTs | water/toluene | 25 | 0.1 | 99.9% | 5000.0 | - | ^[3]^ |
| PVDF-A_0.9_B_0.2_ | water/toluene | 296 | 1 | 97.0% | 1769.0 | 12306.3 | ^[4]^ |
| P(St-D-T)/PEI | water/toluene | 5000 | 0.4 | 97.4% | 8363.0 | - | ^[5]^ |
| PDA/ACNTs@TPU | water/toluene | 172 | 0.2 | 98.9% | 6652.0 | 15671.1 | ^[6]^ |
| Ag@PDA@NM | water/toluene | 120 | 0.9 | 99.2% | 1700.0 | 18082.5 | ^[7]^ |
| HTPVDF | water/hexane | 125 | 0.2 | 99.2% | 536.0 | 55408.7 | ^[8]^ |
| PVC sponge | water/hexane | 2500 | 1 | 99.0% | 161.5 | - | ^[9]^ |
| HI/HO | water/  n-octane | 450 | 0.12 | 99.6% | 2993.0 | - | ^[10]^ |
| PLA-PF-AS | water/  n-octane | / | 0.1 | 99.2% | 13818.8 | - | ^[11]^ |
| PDA/PET/PVDF | water/  chloroform | 300 | 1 | 99.4% | 4475.0 | 9387.3 | ^[12]^ |
| modified PET TeM | water/  dimethylene chloride | 120 | 0.3 | >99.0% | 4316.9 | 21157.3 | ^[13]^ |
| CuBTCFFM | Water/dimethylene chloride | 975 | 0.5 | 99.4% | 8886.4 |  | This work |
| CuBTCFFM | water/toluene | 975 | 0.5 | 98.9% | 9570.7 |  | This work |
| CuBTCFFM | water/hexane | 975 | 0.5 | 99.0% | 17344.2 |  | This work |
| CuBTCFFM | Water/dimethylene chloride | 136 | 0.5 | 99.4% | 17593.3 |  | This work |
| CuBTCFFM | water/toluene | 105 | 0.5 | 98.9% | 19672.2 |  | This work |
| CuBTCFFM | water/hexane | 105 | 0.5 | 99.0% | 60854.3 |  | This work |

-: The thickness of the membranes is not in the fitted data range in this study.


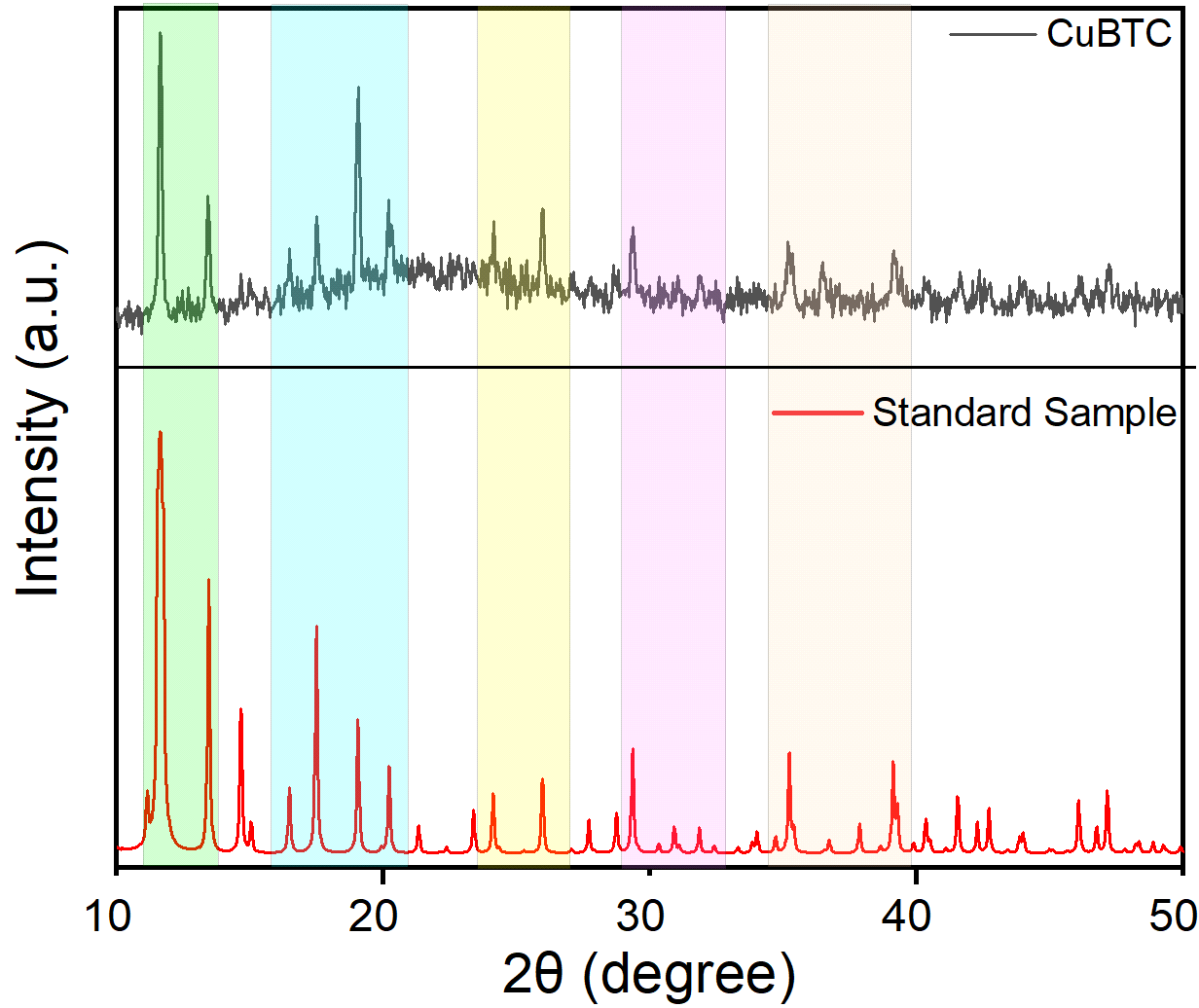


**Figure S1.** XRD patterns of CuBTC.


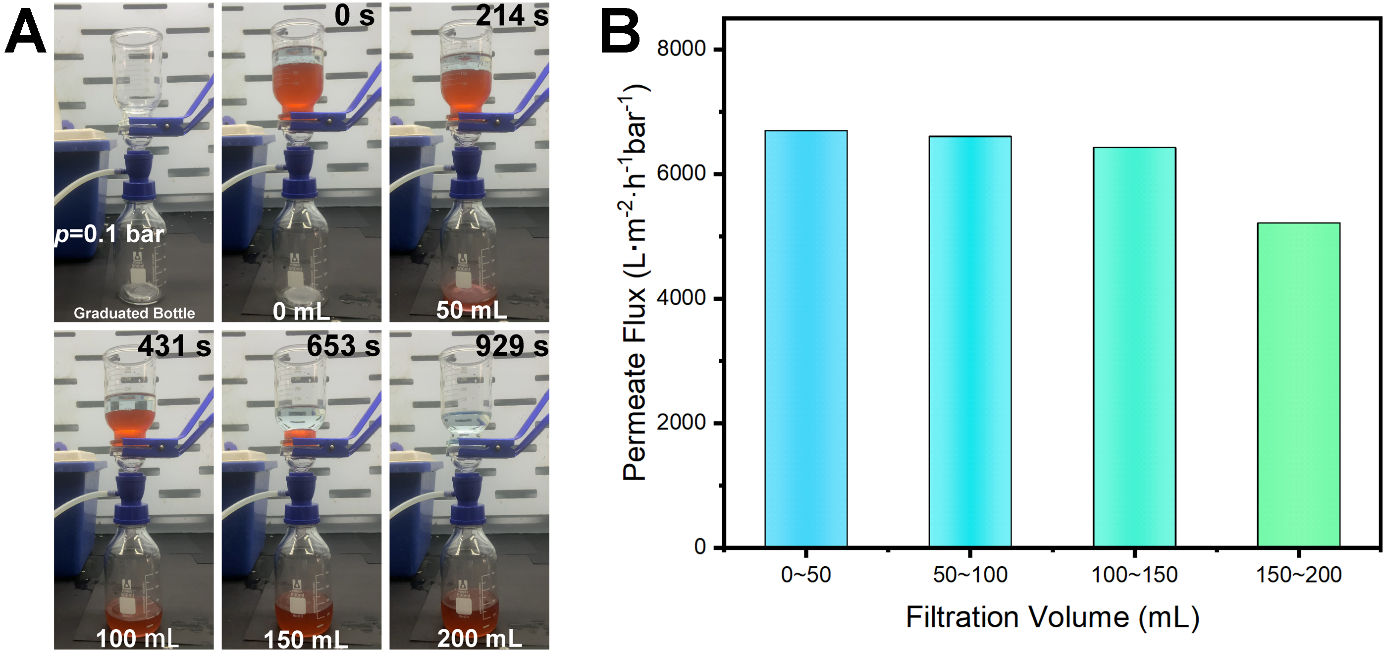


**Figure S2.** (A) Measuring device for the average continuous flux variation during oil-water separation in CuBTCFFM. (B) Average continuous flux variation of CuBTCFFM.


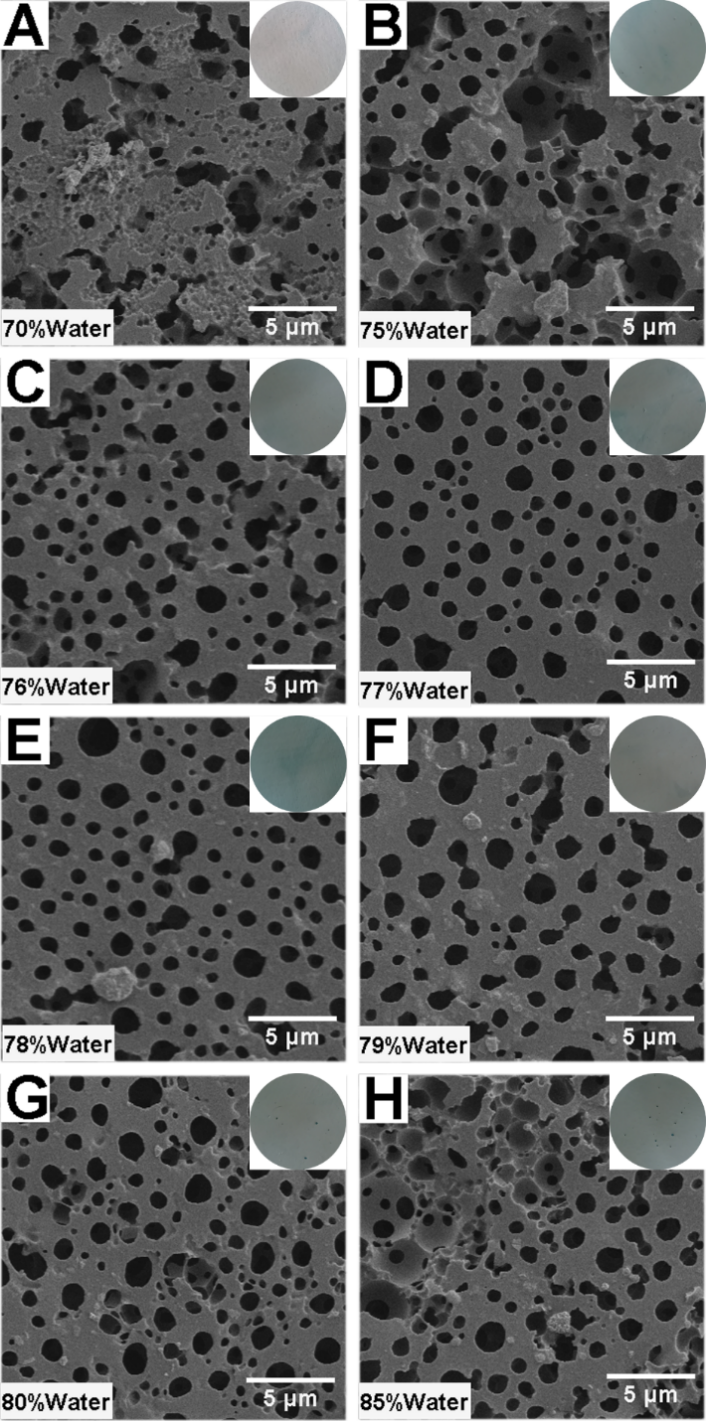


**Figure S3.** SEM images of CuBTCFFMs prepared with different water-oil ratios.


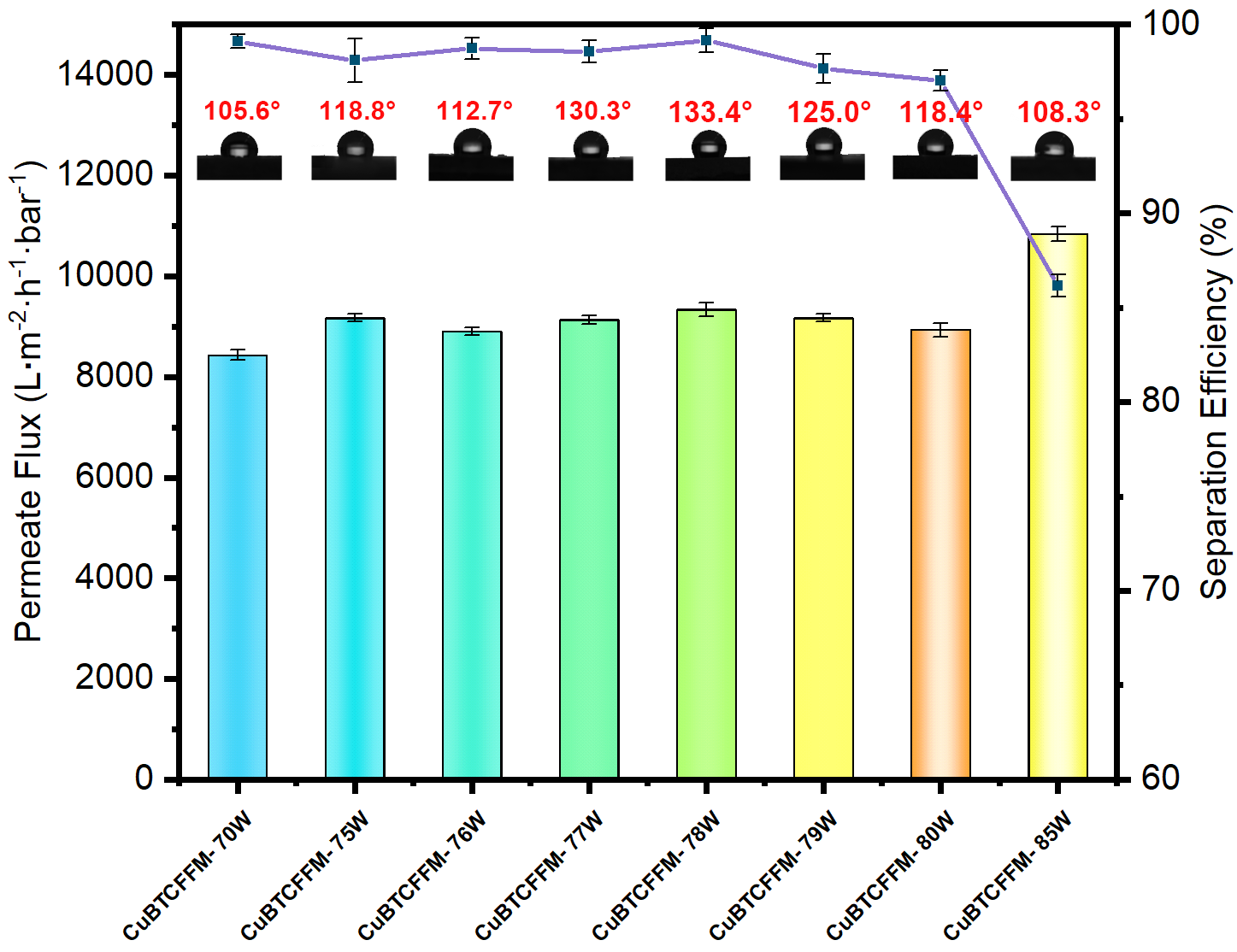


**Figure S4.** Permeate flux (colored bars), separation efficiency (filled symbols) of dichloromethane solvent from water, and water contact angle of CuBTCFFMs prepared with different water-oil ratios.


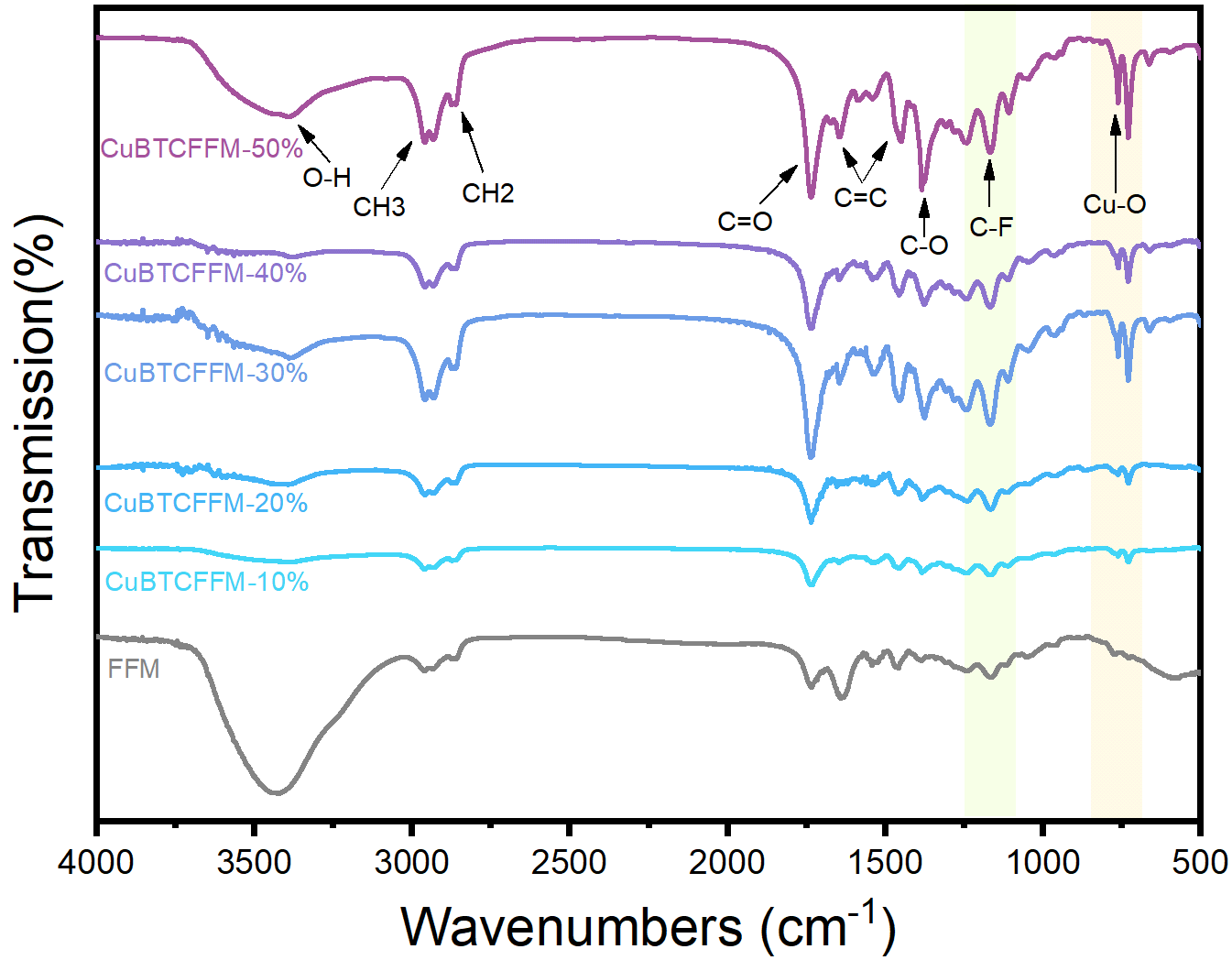


**Figure S5.** The ATR-FTIR spectra of the CuBTCFFMs doped with different CuBTC content.


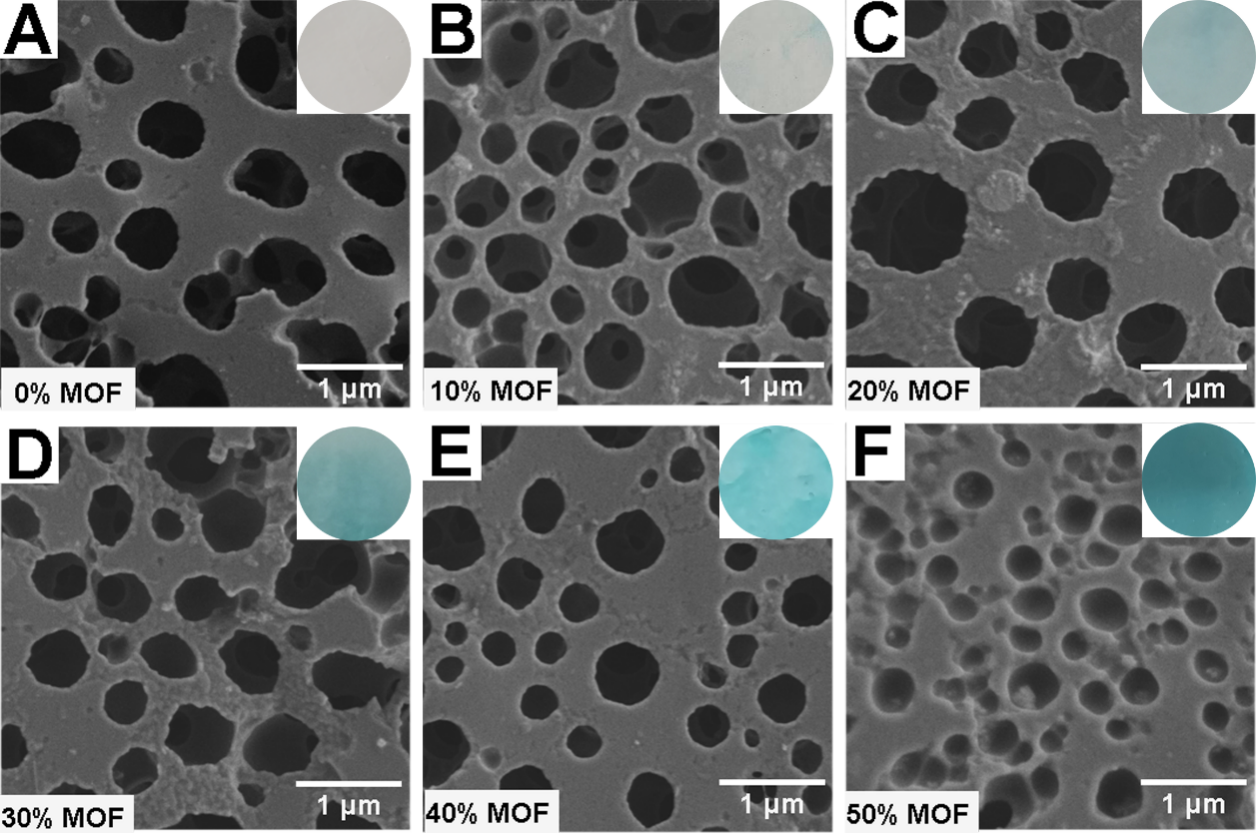


**Figure S6.** SEM images of CuBTCFFMs doped with different CuBTC contents.


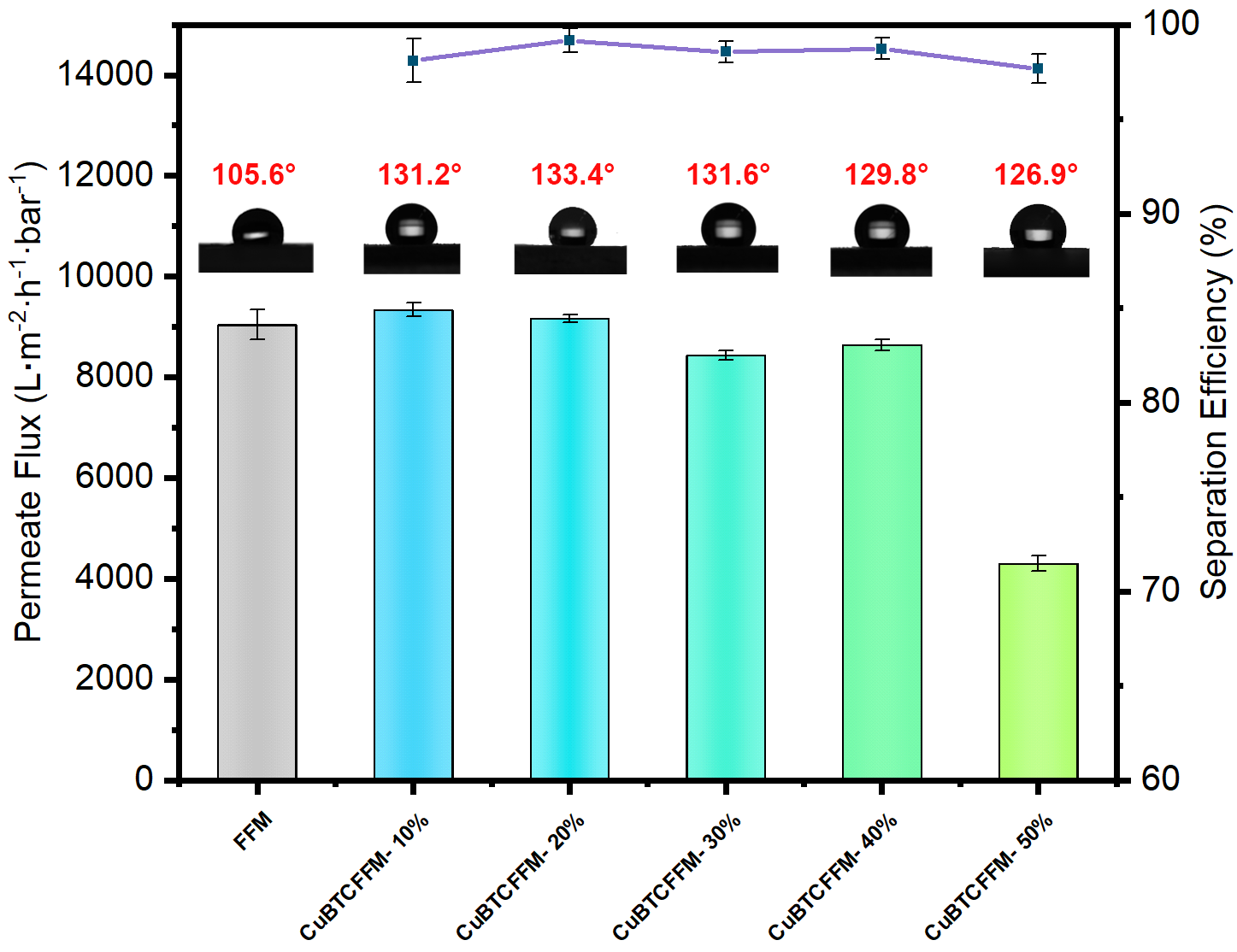


**Figure S7.** Permeate flux (colored bars), separation efficiency (filled symbols) of dichloromethane from water, and water contact angle of CuBTCFFMs doped with different CuBTC content.


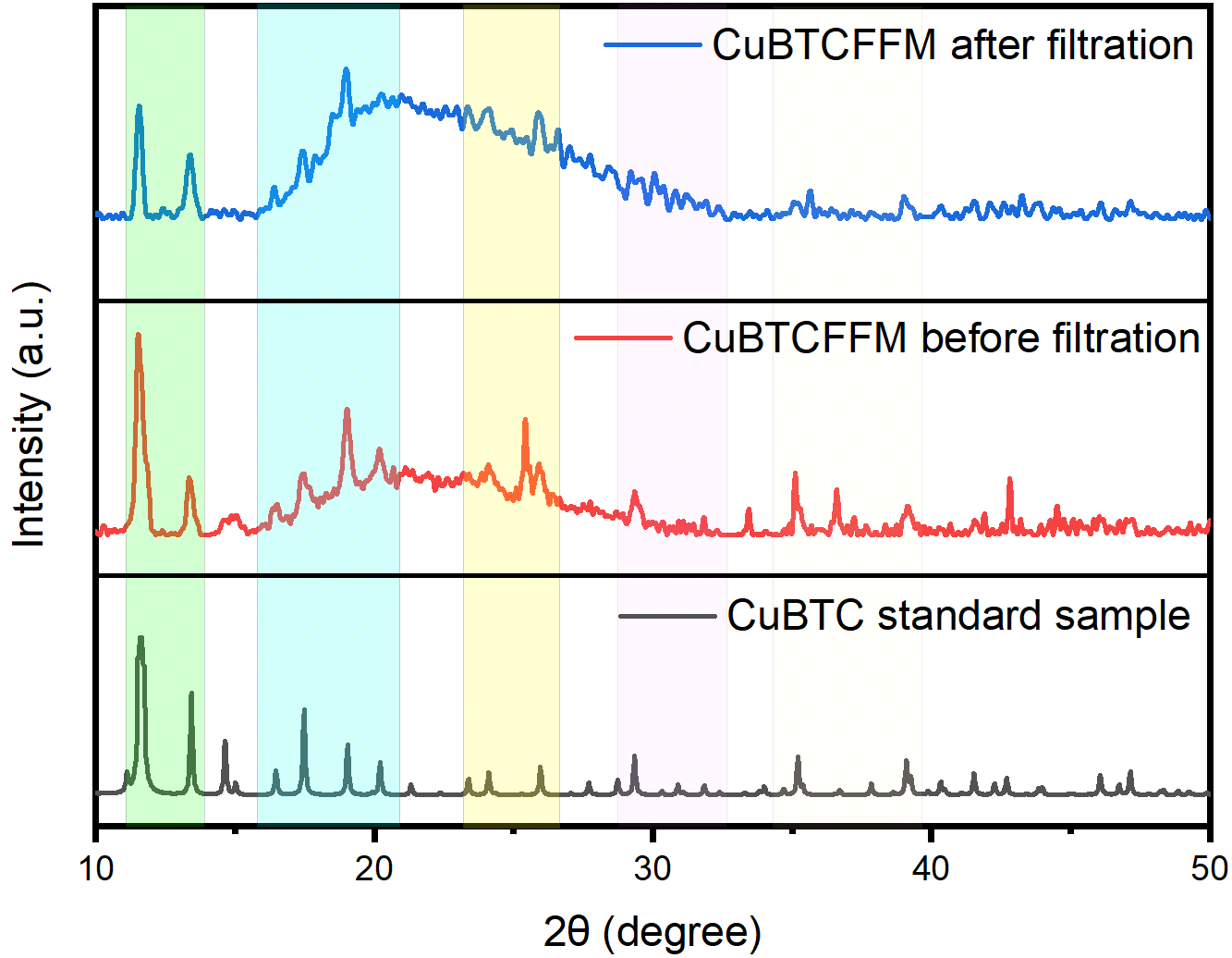


**Figure S8.** XRD patterns of CuBTCFFM before and after oil-water separation.


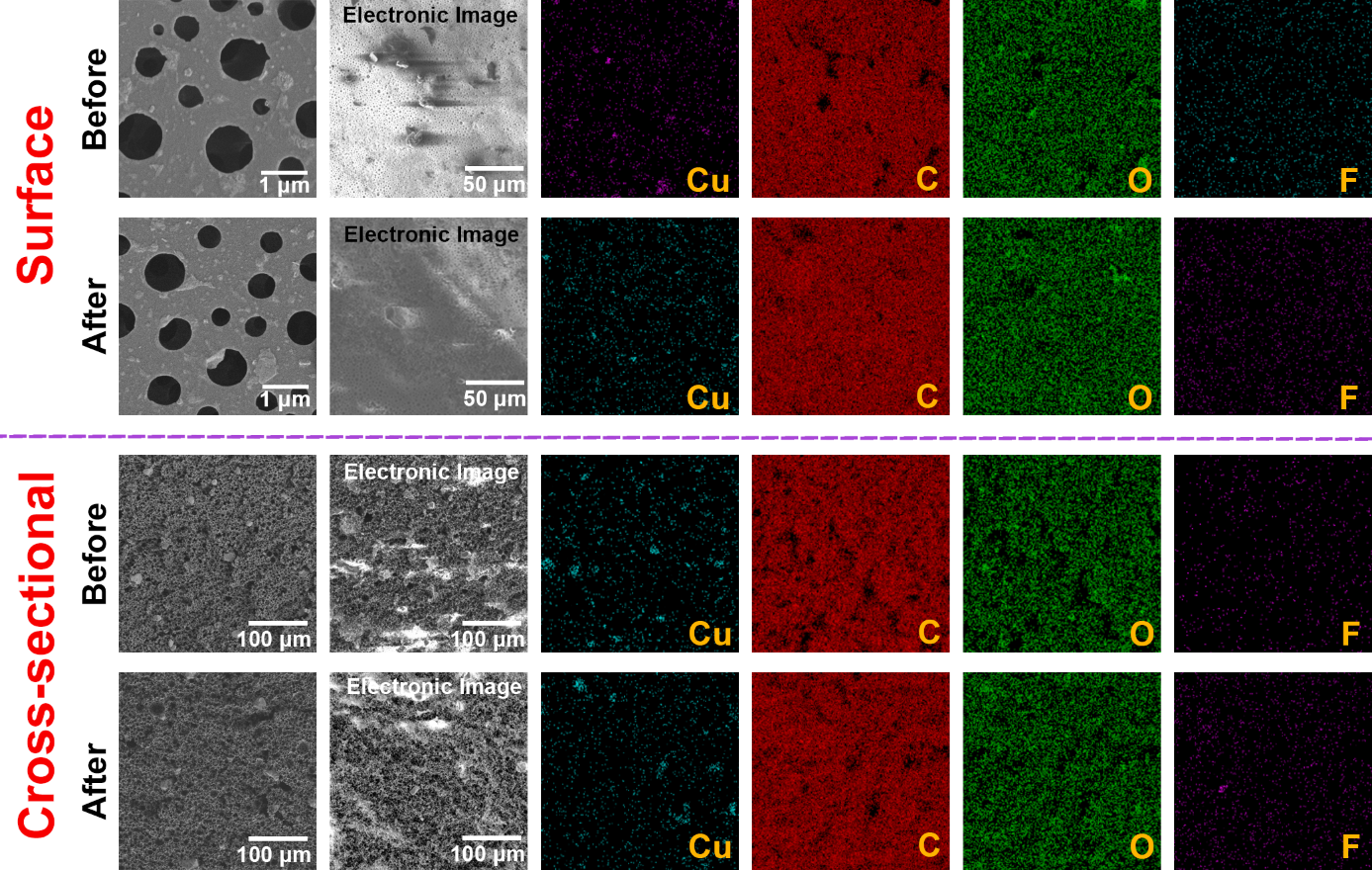


**Figure S9.** SEM images and EDS analysis of CuBTCFFM (surface and cross-sectional) before and after oil-water separation.


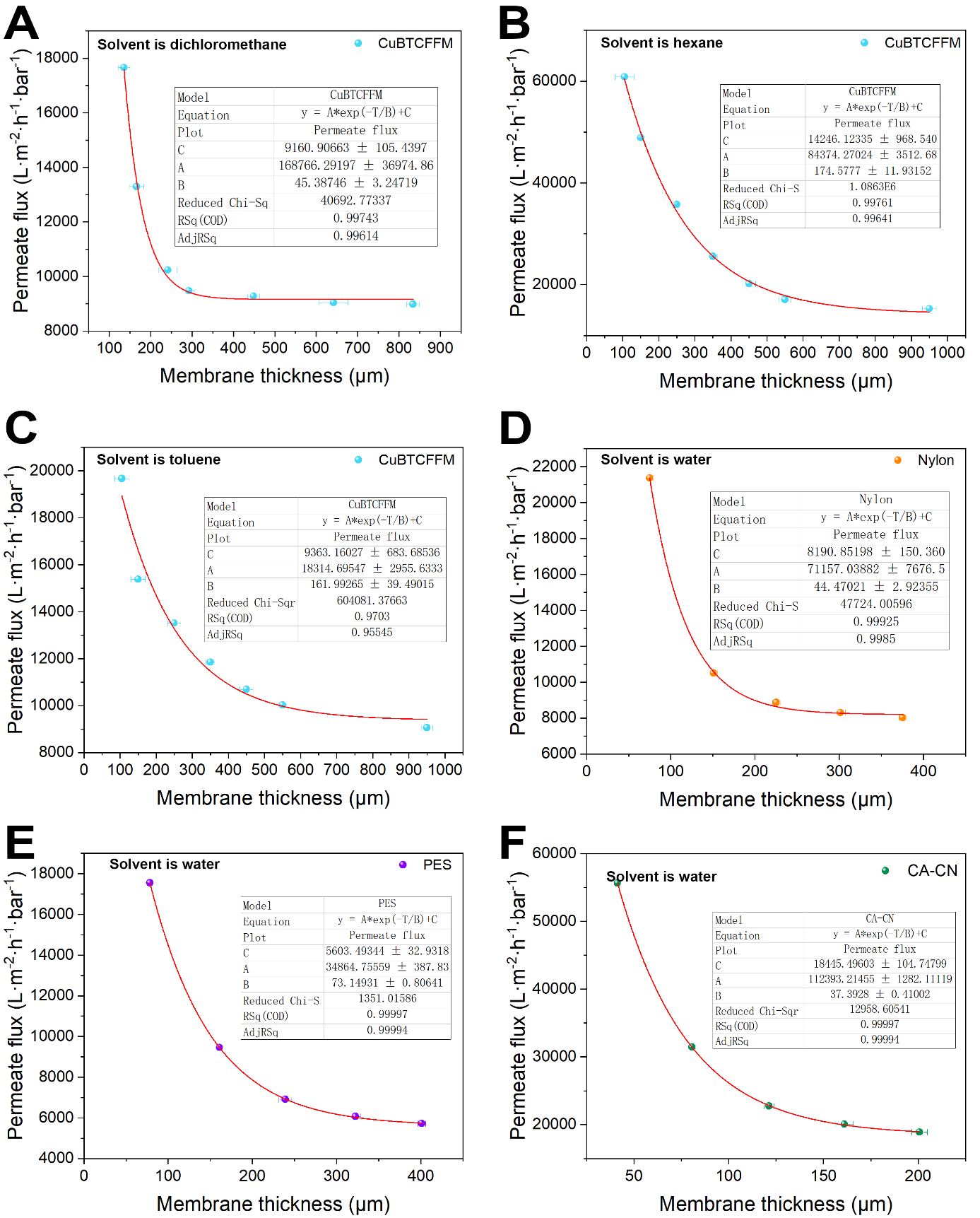


**Figure S10.** Permeate flux for membranes of varying thickness: (A) CuBTCFFM (The solvent is dichloromethane), (B) CuBTCFFM (The solvent is hexane) and (C) CuBTCFFM (The solvent is toluene), (D) Nylon membrane, (E) Polyether sulfone membrane (PES), (F) Cellulose acetate membrane (CA-CN).

Furthermore, the membrane prepared without the addition of TFEMA was discussed (**Figure S12**), wherein the membrane contains CuBTC and EHA (CuBTCEFM). It was observed that the membrane lacked the requisite hydrophobicity to effectively separate oil and water. This is attributed to the absence of fluorine, which is necessary to enhance the membrane's separation efficiency. The interaction energy simulation experiment also proves that CuBTCEFM has no obvious hydrophobicity. Therefore, it was concluded that fluorine monomer must be incorporated to achieve the desired separation performance.


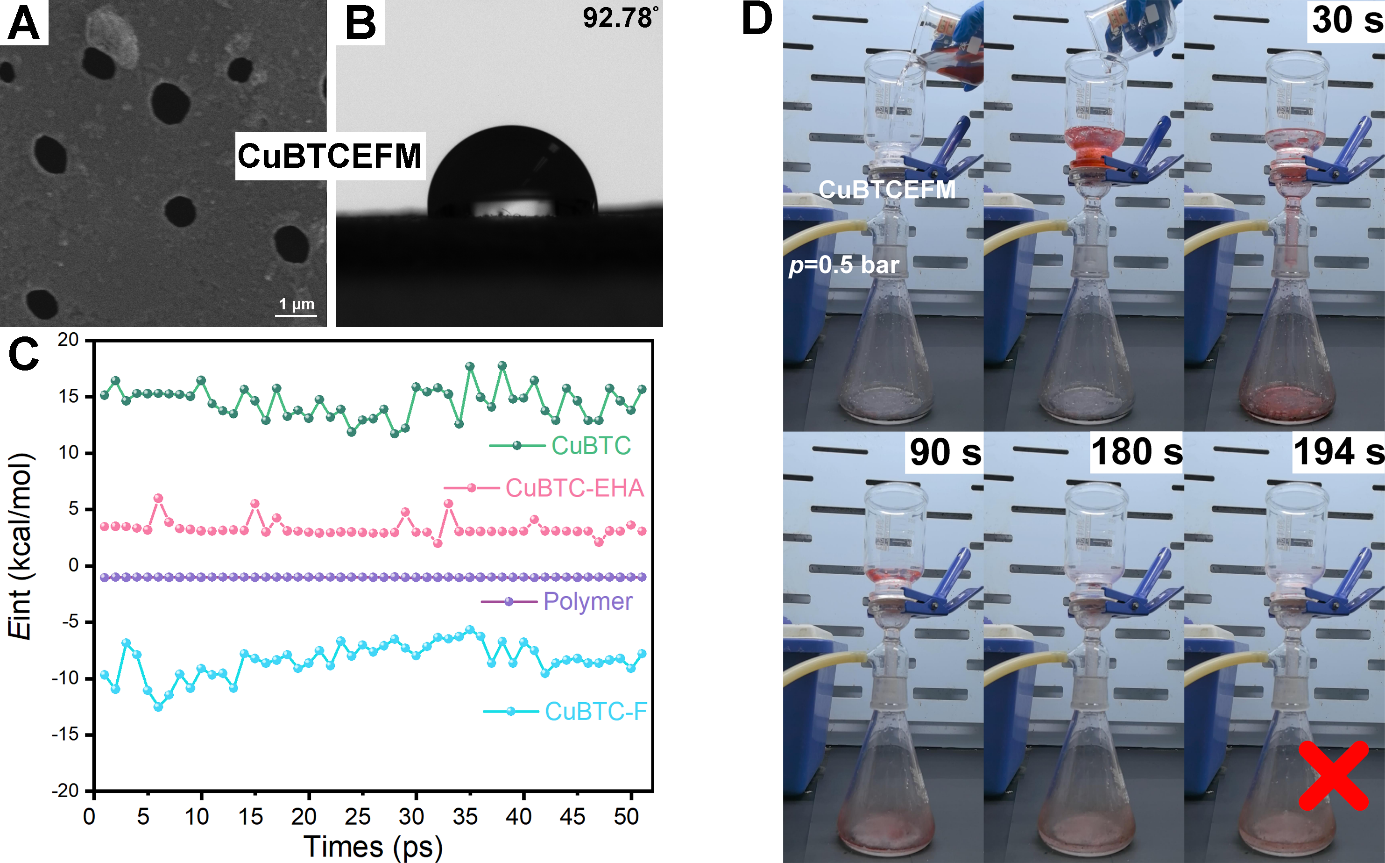


**Figure S11.** (A) SEM image of the surface, (B)water contact angle, and (C) interaction energy simulation (pink) of CuBTCEFM, (D) experimental images of CuBTCEFM for different separation stages of oil-water mixtures.


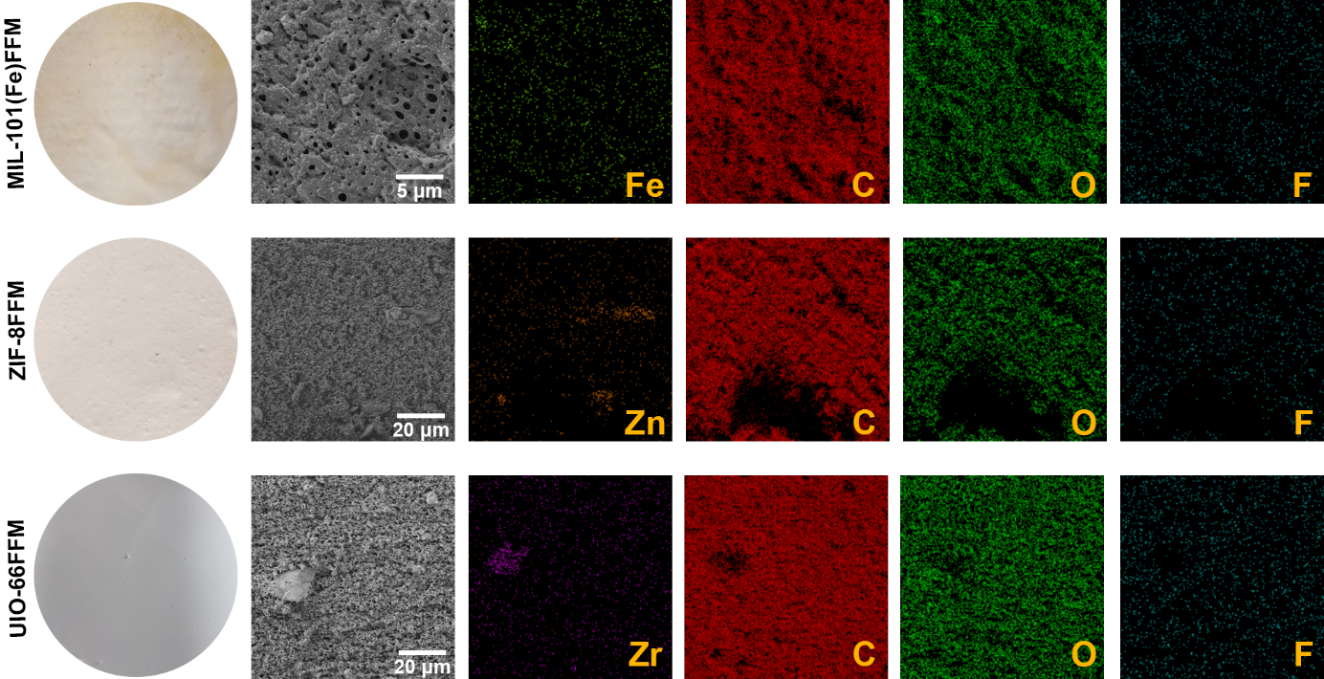


**Figure S12.** SEM images and EDS analysis of MIL-101(Fe)FFM, ZIF-8FFM, and UiO-66FFM doped with different MOFs prepared by HIPE template method.

**References**

[1] J. Yuan, W. Chen, X. Tan, W. Yang, D. Yang, H. Yu, B. Zhou, B. Yang, *Journal of Materials in Civil Engineering* **2021**, 33, 04021117.

[2] I. Prasanthi, B. Rani Bora, K. Raidongia, K. K. R. Datta, *Sep. Purif. Technol.* **2022**, 301, 122049.

[3] J. Gu, P. Xiao, J. Chen, F. Liu, Y. Huang, G. Li, J. Zhang, T. Chen, *J. Mater. Chem. A* **2014**, 2, 15268.

[4] J.-H. Zuo, Y.-H. Gu, C. Wei, X. Yan, Y. Chen, W.-Z. Lang, *J Membrane Sci.* **2020**, 595, 117475.

[5] H. Huang, Y. Cai, C. Zhao, Z. Chen, Z. Liao, H. Xie, H. Li, D. Xiang, Y. Wu, J. Cheng, D. Li, *Sep. Purif. Technol.* **2024**, 339, 126691.

[6] X. Huang, Z. Wu, S. Zhang, W. Xiao, L. Zhang, L. Wang, H. Xue, J. Gao, *J Hazard. Mater.* **2022**, 429, 128250.

[7] L. Zheng, H. Li, X. Lai, W. Huang, Z. Lin, X. Zeng, *J Membrane Sci.* **2022**, 642, 119995.

[8] Y. Ding, N. Qiu, J. Wang, Z. Yang, F. Liu, C. Y. Tang, *J Membrane Sci.* **2023**, 684, 121820.

[9] D. Lang, G. Liu, R. Wu, G. Chen, C. Zhang, C. Yang, W. Wang, J. Wang, J. Fu, *Chem. Eng. J.* **2023**, 471, 144752.

[10] X. Cheng, Y. Ye, Z. Li, X. Chen, Q. Bai, K. Wang, Y. Zhang, E. Drioli, J. Ma, *Acs Nano.* **2022**, 16, 4684.

[11] Y. Ye, T. Li, Y. Zhao, J. Liu, D. Lu, J. Wang, K. Wang, Y. Zhang, J. Ma, E. Drioli, X. Cheng, *Sep. Purif. Technol.* **2023**, 317, 123885.

[12] Q. Xiong, H. Chen, Q. Tian, X. Yue, F. Qiu, T. Zhang, A.-B. Wang, *J Environ. Chem. Eng.* **2022**, 10, 108459.

[13] Z. Guo, Y. Wang, Z. Liang, Z. Zhang, J. Xie, X. Gui, B. Hou, D. Mo, L. Lu, H. Yao, *J Water Process Eng.* **2023**, 54, 103997.
